# The amygdala NT3-TrkC pathway underlies inter-individual differences in fear extinction and related synaptic plasticity

**DOI:** 10.1038/s41380-024-02412-z

**Published:** 2024-01-17

**Authors:** Gianluca Masella, Francisca Silva, Elisa Corti, Garikoitz Azkona, Maria Francisca Madeira, Ângelo R. Tomé, Samira G. Ferreira, Rodrigo A. Cunha, Carlos B. Duarte, Mónica Santos

**Affiliations:** 1 CNC – Center for Neuroscience and Cell Biology, University of Coimbra, Coimbra, Portugal; 2 Institute of Interdisciplinary Research, University of Coimbra (iiiUC), Coimbra, Portugal; 3 Department of Basic Psychological Processes and Their Development, School of Psychology, University of the Basque Country (UPV/EHU), San Sebastian, Spain; 4 Department of Life Sciences, University of Coimbra, Coimbra, Portugal; 5 Faculty of Medicine, University of Coimbra, Coimbra, Portugal; 6 Centre for Innovative Biomedicine and Biotechnology (CIBB), University of Coimbra, Coimbra, Portugal

**Keywords:** Neuroscience, Physiology

## Abstract

Fear-related pathologies are among the most prevalent psychiatric conditions, having inappropriate learned fear and resistance to extinction as cardinal features. Exposure therapy represents a promising therapeutic approach, the efficiency of which depends on inter-individual variation in fear extinction learning, which neurobiological basis is unknown. We characterized a model of extinction learning, whereby fear-conditioned mice were categorized as extinction (EXT)-success or EXT-failure, according to their inherent ability to extinguish fear. In the lateral amygdala, GluN2A-containing NMDAR are required for LTP and stabilization of fear memories, while GluN2B-containing NMDAR are required for LTD and fear extinction. EXT-success mice showed attenuated LTP, strong LTD and higher levels of synaptic GluN2B, while EXT-failure mice showed strong LTP, no LTD and higher levels of synaptic GluN2A. Neurotrophin 3 (NT3) infusion in the lateral amygdala was sufficient to rescue extinction deficits in EXT-failure mice. Mechanistically, activation of tropomyosin receptor kinase C (TrkC) with NT3 in EXT-failure slices attenuated lateral amygdala LTP, in a GluN2B-dependent manner. Conversely, blocking endogenous NT3-TrkC signaling with TrkC-Fc chimera in EXT-success slices strengthened lateral amygdala LTP. Our data support a key role for the NT3-TrkC system in inter-individual differences in fear extinction in rodents, through modulation of amygdalar NMDAR composition and synaptic plasticity.

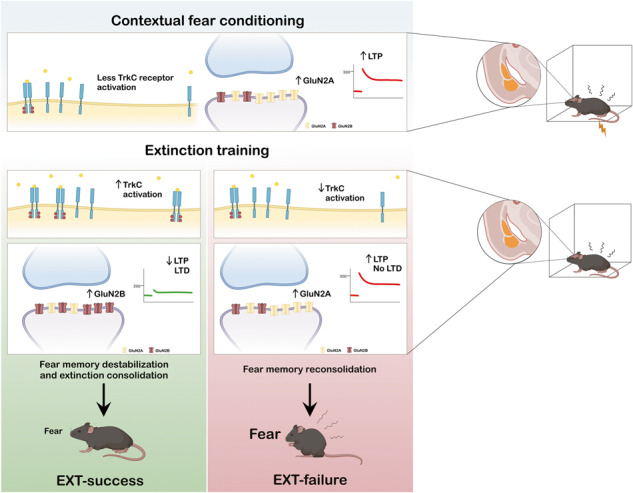

## Introduction

Fear-related disorders gather some of the most commonly diagnosed psychiatric conditions including panic disorder (PAND), several phobias and posttraumatic stress disorder (PTSD). With a high prevalence worldwide, these disorders are highly disruptive to the professional and social life of afflicted individuals and represent considerable governmental and societal costs [reviewed in [1]. Patients in this group of disorders show impaired associative learning of contextual and sensory cues related to their ‘object’ of fear [2, 3], resulting in inappropriate and/or excessive fear, and inability to extinguish maladaptive fear [4]. These impairments in fear extinction negatively impact on the efficiency of exposure therapy, the first line of treatment to patients with anxiety and fear-related disorders [5, 6]. Indeed, inter-individual variation in fear extinction learning is predictive of exposure therapy outcome [7, 8]. We are still lacking mechanism-based therapeutic approaches for those patients with impaired extinction.

Pavlovian fear conditioning and extinction represent a simple, yet robust paradigm to investigate the neural substrates and molecular machinery of acquisition and extinction of learned fear. This model has been instructive to identify the wide network of brain regions that are recruited in the processing of learned fear. The amygdala is the core brain region in the orchestration of fear response, which in turn is modulated by inputs from the hippocampus, conveying information about the context, and from the medial prefrontal cortex (mPFC), providing top-down control [9–12]. Within the amygdala, two distinct, yet interconnected, populations of glutamatergic neurons - fear neurons and extinction neurons – form opposite circuits that regulate fear states [13–16]. It is known that extinction does not erase the previously acquired fear memories, or the underlying microcircuit; instead, at every conditioned stimulus (CS) presentation, the two microcircuits compete to express or suppress fear [15].

Fear learning and extinction are associated with alterations in synaptic strength through long-term potentiation (LTP) and depression (LTD) of synaptic activity [reviewed in [17], which are considered the neurophysiological basis of learning and memory [18–20]. Accordingly, LTP and LTD at amygdalar synapses are believed to underlie acquisition *versus* extinction of learned fear by acting during consolidation and/or reconsolidation of these memories [21, 22]. Indeed, context conditioning induces a selective increase in synaptic strength of context-responding basolateral amygdala (BLA) neurons [23]. On the other hand, extinction learning was found to induce LTD at lateral amygdala (LA) synapses [22]. Interestingly, extinction of both auditory and contextual fear memories reverses the conditioning-induced potentiation of BLA neurons [23, 24].

Amygdalar glutamate receptors play a pivotal role in both LTP and LTD processes that underlie fear conditioning and extinction. In particular, GluN2A- and GluN2B-containing NMDA receptors (NMDAR) play opposite roles in fear processing and amygdalar synaptic plasticity. GluN2A is necessary for the induction of LTP in the amygdala and is required for the acquisition of conditioned fear [25]. Fear learning induces the recruitment of GluN2A-containing NMDAR to BLA synapses, and their abundance is directly correlated to the strength of the fear memory [26]. Moreover, GluN2A-containing NMDAR promote the restabilization of fear memories in reconsolidation [27]. Conversely, GluN2B-containing NMDAR are responsible for amygdalar LTD and are required for extinction learning [25], as well as for the destabilization of fear memories made labile by retrieval [27, 28].

Accumulating evidence suggests a role for neurotrophins in the regulation of fear [29–32]. In particular, the neurotrophin 3 (NT3) - tropomyosin receptor kinase C (TrkC) system has been associated to anxiety disorders in human and non-human primates [33–35], and in mouse models of disease [29, 36, 37]. In addition, our recent studies underscored the importance of TrkC signaling in the formation of fear memories in normal physiological conditions [32]. Neurotrophins have a critical role in synaptic plasticity [38–40] prompting them as candidates to mediate the cognitive-emotional regulation of fear [41].

In this study, taking advantage of intrinsic inter-individual variation in fear extinction performance, we have characterized a model to investigate the substrates that support differences between mice that successfully extinguish fear and those that fail. Using this model, we unveiled a role of amygdalar NT3-TrkC system in the regulation of fear extinction. Activation of the NT3-TrkC system results in attenuation of learning-induced LTP, in a GluN2B-dependent mechanism, which seems paramount for successful fear extinction.

## Results

### Inter-individual variation in context fear extinction

**Fig. 1:**
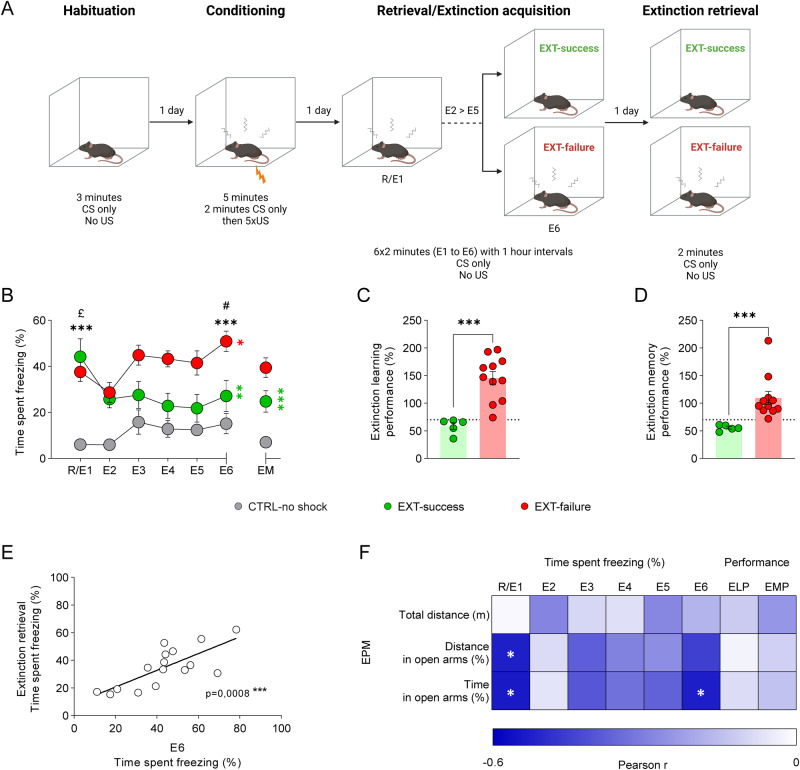
Inter-individual variation in context fear extinction. **A** Schematic representation of the Pavlovian contextual fear conditioning and extinction paradigm to which young adult (8 to 12 weeks old) C56BL/6J male mice were submitted throughout this study. **B** Quantification of the percentage of time spent freezing during fear extinction acquisition and extinction memory retrieval. Fear-conditioned mice were categorized as EXT-success (*n* = 5) or EXT-failure (*n* = 11) according to their extinction learning performance. A CTRL-no shock group was included that received no shocks (*n* = 9). Repeated measures two-way ANOVA with either Sidak or Tukey multiple comparisons test. * CTRL-no shock vs. EXT-failure; £ CTRL-no shock vs. EXT-success; # EXT-success vs. EXT-failure; * EXT-success, R/E1 vs. E6 and R/E1 vs. EM; * EXT-failure, R/E1 vs. E6. **C** Extinction learning performance, expressed as the percentage of freezing levels in E6 relative to freezing in R/E1. The dotted line marks the threshold of 30% reduction from R/E1 used to categorize mice as EXT-success or EXT-failure. *** two-tailed Student’s *t* test. **D** Extinction memory performance, expressed as the percentage of freezing levels in extinction retrieval relative to freezing in R/E1. *** Mann–Whitney U test. **E** Correlation of the freezing levels during the last trial of extinction acquisition (E6) with the freezing levels in extinction retrieval. Pearson r. **F** Correlation matrix of the total distance traveled, distance in open arms and time spent in the open arms in the EPM test with the percentage of time spent freezing during extinction acquisition trials and with ELP and EMP; statistics using Pearson r. CS conditioned stimulus, E1 to E6 extinction trials, EM extinction memory retrieval, ELP extinction learning performance, EMP extinction memory performance, EPM elevated plus maze, R fear retrieval, US unconditioned stimulus.; *^,£,#^*p* ≤ 0.05, **p ≤ 0.01, ***p ≤ 0.001.

Young adult C57BL/6J male mice (*n* = 16) were trained in the contextual fear conditioning (CFC) and extinction (EXT) paradigm (Fig. 1A). A control group that did not receive any shock was also included (CTRL-no shock, *n* = 9). With consecutive shock administrations, conditioned mice showed a progressive increase in the percentage of time spent freezing (Supplementary Fig. S1A) and, when tested 24 h later for fear retrieval showed a proper conditioned fear response (Supplementary Fig. S1B). After fear retrieval, mice were immediately trained in fear extinction acquisition. Here, extinction learning performance was defined for each individual as the ratio (expressed as percentage) between freezing levels in the last trial of extinction acquisition (E6) and those shown in fear memory retrieval/first trial of extinction acquisition (R/E1). Extinction learning performance was used to evaluate extinction and categorize mice as EXT-success (>30% reduction in freezing levels at E6 relative to R/E1) or EXT-failure (<30% or no reduction in freezing).

Throughout extinction session trials (R/E1 to E6), EXT-success animals showed a reduction in freezing to levels comparable to those of CTRL-no shock animals, while EXT-failure mice increased their freezing levels (Fig. 1B, extinction trial x group interaction *F*_(10, 110)_ = 2.862, *p* = 0.003; R/E1 vs. E6, EXT-success *t* = 8.675, *p* = 0.0048; EXT-failure *t* = 3.215, *p* = 0.0454). In particular, in R/E1 trial, both EXT-success and EXT-failure conditioned mice froze significantly more than CTRL-no shock mice (CTRL-no shock vs. EXT-success *t* = 4.618, *p* = 0.012, CTRL-no shock vs. EXT-failure *t* = 6.400, *p* < 0.001, EXT-success vs. EXT-failure *t* = 0.7485, *p* = 0.481), showing the proper formation of a contextual fear memory. By trial E6, EXT-success mice showed freezing levels comparable to those of CTRL-no shock mice, and significantly lower than EXT-failure mice (CTRL-no shock vs. EXT-success *t* = 1.490, *p* = 0.177, CTRL-no shock vs. EXT-failure *t* = 5.727, *p* < 0.001, EXT-success vs. EXT-failure *t* = 2.947, *p* = 0.039). Overall, EXT-success mice showed an average reduction in freezing levels of more than 40% relative to the levels shown in R/E1, as opposed to a 45% increase in freezing levels exhibited by EXT-failure animals (Fig. 1C, *t* = 4.664, *p* < 0.001).

In the extinction memory retrieval phase (EM), EXT-success animals showed statistically significant lower levels of freezing as compared to R/E1 session (Fig. 1B; extinction session x group interaction, *F*_(2, 22)_ = 9.566, *p* = 0.0010; EXT-success, R/E1 vs EM *t* = 4.537, *p* = 0.0005), while EXT-failure animals did not show a decrease in the freezing levels from R/E1 to EM (EXT-failure, R/E1 vs EM *t* = 0.6425, *p* = 0.8943). Extinction memory performance (EMP) was defined as the ratio (expressed as percentage) between freezing levels in extinction retrieval and those shown in R/E1. EXT-success mice showed a better extinction memory performance as compared to EXT-failure animals when tested for extinction retrieval 24 h later. Here, EXT-success animals showed an average 45% reduction in freezing levels relative to the levels shown in R/E1, as opposed an average 9% increase in freezing exhibited by EXT-failure animals (Fig. 1D, U = 0, *p* < 0.001). Importantly, we detected a statistically significant correlation between freezing levels in E6 and freezing levels in the extinction retrieval session (Fig. 1E; R^2^ = 0.5647, *p* < 0.001), suggesting that extinction learning by E6 is predictive of extinction memory performance.

The previous correlation suggests that individual differences in extinction could be a stable trait of the animals, which could be predicted by other behavioral traits. To evaluate this hypothesis, we investigated how fear extinction and ability to learn to extinguish fear memories could be correlated with individual differences in trait anxiety. Basal anxiety-like behavior and exploratory activity were assessed in the open field (OF) and elevated plus maze (EPM) tests, before performance in the CFC and EXT paradigm. In the OF, no differences were observed among EXT-success, EXT-failure and CTRL-no shock mice in the total distance traveled nor in the percentage of time spent or distance traveled in the center of the arena (Supplementary Fig. S1C–E). Also, no differences were found among the different groups in the EPM in the total distance traveled, percentage of open arms time or percentage of open arms distance (Supplementary Fig. S1F–H). We found however that basal anxiety levels are predictive of the strength of the fear memory, but not of learning performance itself. Indeed, a statistically significant negative correlation was found between the percentage of time spent in the open arms (OAT), as well as the distance traveled in the open arms (OAD), and freezing levels in R/E1 and E6 trials of extinction acquisition (Fig. 1F, OAT vs. R/E1, R^2^ = 0.2916, *p* = 0.030; OAT vs. E6, R^2^ = 0.2809, *p* = 0.035; OAD vs. R/E1, R^2^ = 0.2809, *p* = 0.035; OAD vs. E6, R^2^ = 0.2025, *p* = 0.077), but not with extinction learning performance and extinction memory performance (Fig. 1F, OAT vs. ELP, R^2^ = 0, 0064, *p* = 0.757; OAT vs. EMP, R^2^ = 0.0225, *p* = 0.590; OAD vs. ELP, R^2^ = 0, 0009, *p* = 0.913; OAT vs. EMP, R^2^ = 0.0121, *p* = 0.679).

#### Successful extinction learning is associated with weak LTP and strong LTD at LA synapses

**Fig. 2:**
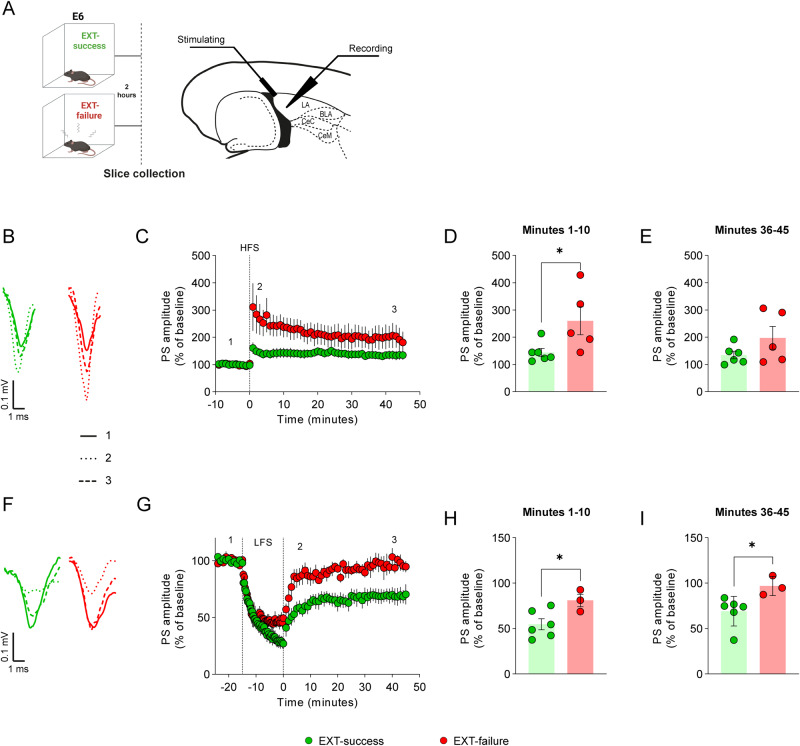
EXT-success mice show attenuated LTP and increased LTD in LA synapses. **A** Schematic representation of experimental conditions and localization of stimulating and recording electrodes in horizontal slices containing the LA. **B**–**E** LTP was induced ex-vivo in the LA of EXT-success (*n* = 6) and EXT-failure (*n* = 5) brain slices with a HFS protocol (three 1 s duration trains of 100 Hz pulses, with 5 s inter-train interval; vertical dashed line). **B** Representative traces for EXT-success and EXT-failure slices at baseline (^__^), upon 10 min (^…^) and upon 45 min (---) after HFS. **C** Time course of LTP recorded for 45 min following LTP induction. PS amplitude was averaged for the (**D**) first 10 min and (**E**) last 10 min of recordings following LTP induction. **F**–**I** LTD was induced ex-vivo in the LA of EXT-success (*n* = 6) and EXT-failure (*n* = 3) brain slices with a low-frequency stimulation protocol (900 stimuli at 1 Hz; between the two vertical lines). **F** Representative traces for EXT-success and EXT-failure slices at baseline (^__^), upon 10 min (^…^) and 45 min (---) of LTD induction with the LFS train. **G** Time course of LTD recorded for 45 min following induction. PS amplitude was averaged for the (**H**) first 10 min and (**I**) last 10 min of recordings. **D**, **E**, **H**, **I** Two-tailed Student’s *t* test and Mann–Whitney U test. BLA basolateral amygdala, Ce central amygdala, E6 extinction trial 6, HFS high-frequency stimulation, LA lateral amygdala, LFS low-frequency stimulation, PS population spikes. *p ≤ 0.05.

Synaptic plasticity in the LA represents a cellular correlate of fear conditioning and extinction [24, 42]. In particular, fear conditioning has been associated with LTP at LA synapses [43], while fear extinction is associated with LTD in the same region [22]. Here, we investigated whether differences in fear extinction learning, as those shown by EXT-success and EXT-failure mice, are supported by differences in LA synaptic plasticity. Fear-conditioned mice were trained in the EXT paradigm and categorized as EXT-success or EXT-failure, according to their extinction learning performance (Supplementary Fig. S2A, B). Horizontal brain slices including the amygdala were obtained from EXT-success (*n* = 6 slices from 6 mice) and EXT-failure (*n* = 5 slices from 5 mice) mice at extinction consolidation window (two hours after E6 trial), and electrical stimulation and electrophysiological recordings were performed in the LA (Fig. 2A).

No differences were observed in the input/output (I/O) profiles between EXT-success and EXT-failure slices (Supplementary Fig. S2C), demonstrating that the two groups showed a similar LA basal excitability. High-frequency stimulation (HFS) protocol (three trains of 100 Hz pulses, 1 s duration, 5 s intervals) successfully induced LTP in LA synapses of both EXT-success and EXT-failure slices, as shown by an increase in population spikes (PS) amplitude both in the first 10 min post-HFS and in the last 10 min of recordings, as compared to baseline (Fig. 2C; EXT-success, baseline vs. min 1–10, *t* = 2.948 *p* = 0.0146, baseline vs. min 36–45, *t* = 2.513 *p* = 0.0307; EXT-failure, baseline vs. min 1–10, *t* = 3.141 *p* = 0.0138, baseline vs. min 36–45, *t* = 2.313 *p* = 0.0495). However, slices from EXT-success mice showed a statistically significant weaker potentiation than those from EXT-failure mice in the first 10 min after HFS (Fig. 2D, *t* = 2.383, *p* = 0.0410). Differences in LTP were lost in the last 10 min of recordings (Fig. 2E, *t* = 1.532, *p* = 0.1592), suggesting that alterations in the LTP induction phase underlie differences in EXT-success and EXT-failure behavioral performance.

Low-frequency stimulation (LFS, 900 stimuli at 1 Hz) successfully induced LTD in EXT-success slices, causing a decrease in PS amplitude both in the first 10 min post-LFS and in the last 10 min of recordings, as compared to baseline (Fig. 2G; EXT-success, baseline vs. min 1–10, *t* = 7.391 *p* < 0.001, baseline vs. min 36–45 *t* = 4.611 *p* = 0.001). The same protocol did not induce LTD in EXT-failure slices (Fig. 2G; EXT-failure, baseline vs. min 1–10, *t* = 2.764 *p* = 0.0506, baseline vs. min 36–45 *t* = 0.6347 *p* = 0.6347). Brain slices from EXT-success mice showed lower PS amplitude than those from EXT-failure mice in the first 10 min post-LFS (Fig. 2H, *t* = 2.614, *p* = 0.0347; EXT-success *n* = 6 slices from 5 mice, EXT-failure *n* = 3 slices from 3 mice), which was maintained in the last 10 min of recordings (Fig. 2I, U = 0, *p* = 0.0238).

Taken together with the behavioral data, these results show that individuals with naturally occurring differences in fear extinction have different synaptic plasticity properties in the LA.

#### Amygdalar GluN2A- and GluN2B-containing NMDA receptors underlie group differences in fear extinction

**Fig. 3:**
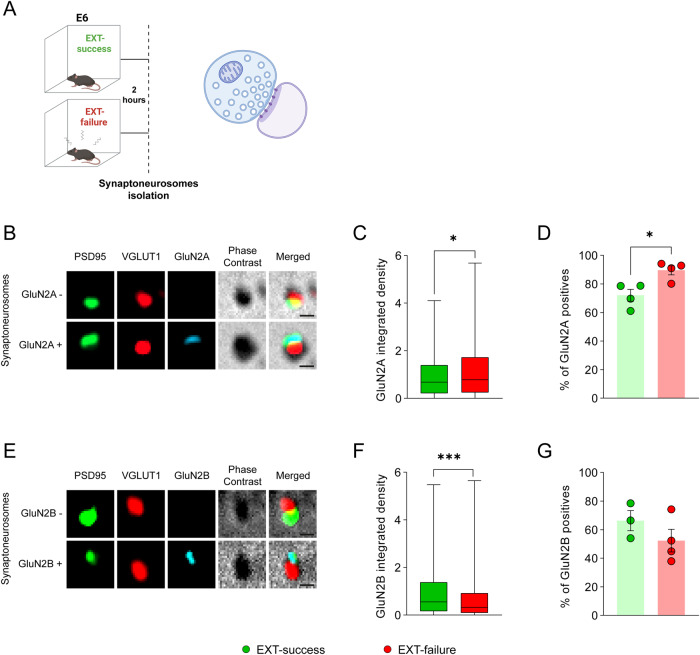
Differential surface density of GluN2A and GluN2B subunits of NMDA receptors in amygdala synaptoneurosomes from EXT-success and EXT-failure mice. **A** Schematic representation of experimental conditions and biologic material used. **B**, **E** Representative images of synaptoneurosomes isolated from the amygdalae of EXT-success and EXT-failure mice, live stained for GluN2A and GluN2B subunits of NMDA receptors. Synaptoneurosomes were identified with co-staining against the postsynaptic marker PSD95 and the presynaptic marker VGlut1 and inspection of intact membranes by phase contrast. **C**, **F** Integrated density of GluN2A and GluN2B signal was quantified in synaptoneurosomes isolated from the amygdalae of EXT success (GluN2A *n* = 631, GluN2B *n* = 529) and EXT-failure (GluN2A *n* = 612; GluN2B *n* = 518) mice. **D**, **G** The percentage of GluN2A- and GluN2B-positive amygdala synaptoneurosomes was calculated for EXT-success and EXT-failure animals (*n* = 4 independent experiments). **C**, **F** Mann–Whitney U test, (**D**, **G**) two-tailed Student’s *t* test. E6 extinction trial 6, GluN2A subunit 2A of NMDA receptor, GluN2B subunit 2B of NMDA receptor, PSD95 postsynaptic density 95, VGluT1 vesicular glutamate transporter 1. Scale bar 0.5 µm. *p ≤ 0.05, ***p ≤ 0.001.

At the molecular level, amygdalar GluN2A- and GluN2B-containing NMDAR play separate roles in fear conditioning and extinction, and in the underlying synaptic plasticity events. In particular, GluN2A has been associated to fear learning and LTP, while GluN2B has been associated to fear extinction and LTD [25, 44]. Moreover, while GluN2A/B are related with the induction phase of LTP and LTD, AMPA receptors are linked to the maintenance of basal and potentiated synaptic transmission [45, 46]. We investigated whether changes in the surface density of glutamate receptors stand on the basis of behavioral and cellular differences between EXT-success and EXT-failure animals. To address this question, fear-conditioned mice were trained in the EXT paradigm and categorized as EXT-success or EXT-failure, according to their extinction learning performance (Supplementary Fig. S3A, B). Two hours after E6 trial, at extinction consolidation window, amygdalae synaptoneurosomes were isolated from EXT-success and EXT-failure mice (Fig. 3A) and stained for surface GluN2A- and GluN2B-containing NMDAR (Fig. 3B, E) and GluA1- and GluA2-containing AMPA receptors (AMPAR) (Supplementary Fig. S3C, F).

We observed that, as compared to EXT-success mice, synaptoneurosomes isolated from the amygdalae of EXT-failure animals showed a higher intensity of GluN2A signal (Fig. 3C, U = 180587, *p* = 0.0482; synaptoneurosomes: EXT-success *n* = 631, EXT-failure *n* = 612) and a higher percentage of GluN2A-positive synaptoneurosomes (Fig. 3D, *t* = 3.312, *p* = 0.0162; synaptoneurosomal preparations: EXT-success *n* = 4, EXT-failure *n* = 4). In turn, the intensity of GluN2B signal was higher in EXT-success than in EXT-failure amygdalae synaptoneurosomes (Fig. 3F, U = 113552, *p* < 0.001; synaptoneurosomes: EXT-success *n* = 529, EXT-failure *n* = 518). No differences were observed in the percentage of GluN2B-positive synaptoneurosomes isolated from the amygdalae of EXT-success and EXT-failure animals (Fig. 3G, *t* = 1.268, *p* = 0.2607; synaptoneurosomal preparations: EXT-success *n* = 3, EXT-failure *n* = 4).

For AMPA receptors, we did not observe any difference in the percentage of GluA1- and GluA2-positive synaptoneurosomes, nor in the intensity of GluA1 and GluA2 signals, between EXT-success and EXT-failure mice (Supplementary Fig. S3C–H).

Overall, interindividual differences in fear extinction learning evidenced using a within-session contextual fear extinction model are corroborated by cellular and molecular substrates of learning, and add to the pool of tools already available to investigate the neural and molecular mechanisms of extinction.

#### Amygdalar TrkC activation correlates with successful fear extinction

In previous studies, we have shown the involvement of NT3-TrkC pathway in the regulation of fear memory formation and extinction in both pathological and normal physiological conditions [29, 32, 37]. Taking advantage of this previous knowledge, we investigated the role of the NT3-TrkC pathway in our fear extinction model.

**Fig. 4:**
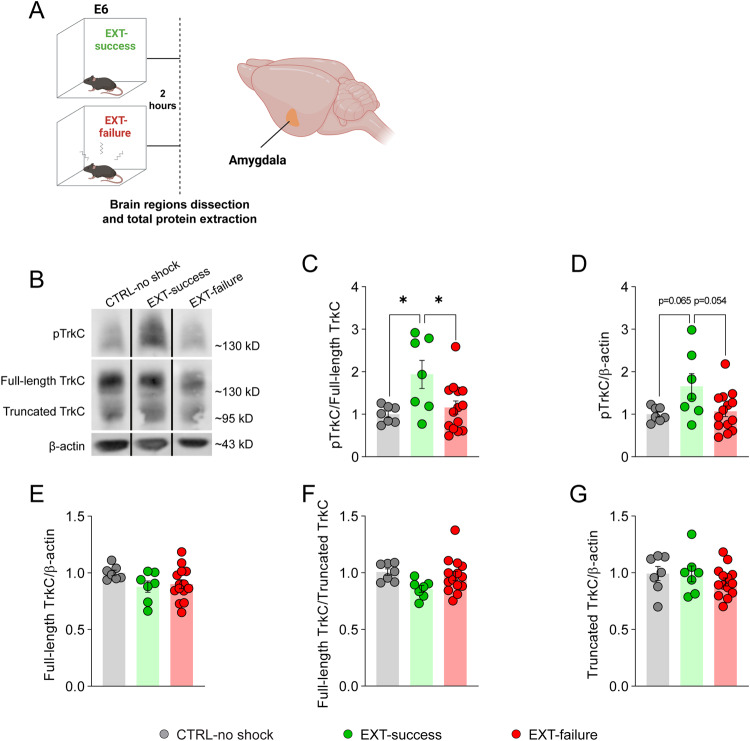
Amygdalar TrkC activation is associated with a successful fear extinction performance. **A** Schematic representation of experimental conditions and dissected brain region. **B** Representative images of western blots for phosphorylated TrkC and total TrkC performed in brain extracts from the amygdala. The panel shows non-contiguous lanes from the same membrane. Quantification of (**C**) relative TrkC activation as measured by pTrkC/full-length TrkC ratio, **(D)** total pTrkC levels, **(E)** total full-length TrkC levels, (**F**) full-length TrkC/truncated TrkC ratio and (**G**) truncated TrkC levels. CTRL-no shock (*n* = 7), EXT-success (*n* = 7) and EXT-failure (*n* = 14) mice. β-actin was used as a loading control. **C**–**G** One-way ANOVA with Tukey’s multiple comparisons test. CTRL control group, E6 extinction trial 6, pTrkC phosphorylated TrkC, TrkC tropomyosin receptor kinase C. *p ≤ 0.05.

Fear-conditioned mice were trained in the EXT paradigm and categorized as EXT-success or EXT-failure, according to their fear extinction learning performance (Supplementary Fig. S4A, B). Two hours after E6 trial, within the extinction consolidation window, mice were killed and the amygdalae, hippocampi, and PFC brain regions were dissected (CTRL-no shock *n* = 7; EXT-success *n* = 7; EXT-failure *n* = 14). Western blots of total protein extracts were performed to measure TrkC activation and density (Fig. 4A, B, Supplementary Fig. S4).

In the amygdala, we observed an increase in relative TrkC activation, as measured by pTrkC/full-length TrkC ratio, in EXT-success mice as compared to CTRL-no shock and EXT-failure mice (Fig. 4C; *F*_(2, 25)_ = 5.121, *p* = 0.0137; CTRL-no shock vs. EXT-success *q* = 4.101, *p* = 0.0202, CTRL-no shock vs. EXT-failure *q* = 0.8053, *p* = 0.8374, EXT-success vs. EXT-failure *q* = 3.931, *p* = 0.0266). Although not statistically significant, the same trend was observed in the total levels of phosphorylated TrkC (Fig. 4D; *F*_(2, 25)_ = 3.685, *p* = 0.040; CTRL-no shock vs. EXT-success *q* = 3.350, *p* = 0.065, CTRL-no shock vs. EXT-failure *q* = 0.4027, *p* = 0.956, EXT-success vs. EXT-failure *q* = 3.466, *p* = 0.054). No differences were observed in the total levels of full-length TrkC (Fig. 4E; *F*_(2, 25)_ = 1.917, *p* = 0.1680).

In the PFC, we observed a decrease in pTrkC/full-length TrkC ratio in EXT-success mice as compared with EXT-failure (Supplementary Fig. S4D). Differences were not due to decreased levels of pTrkC, as no changes were observed in any of the groups (Supplementary Fig. S4E), but to differences in full-length TrkC density, increased in EXT-success as compared with EXT-failure mice (Supplementary Fig. S4F).

In the hippocampus, we did not observe any difference among groups in TrkC activation as measured by pTrkC/full-length TrkC ratio (Supplementary Fig. S4J), total pTrkC levels (Supplementary Fig. S4K) or total TrkC levels (Supplementary Fig. S4L).

The truncated isoform of TrkC lacking the phosphorylation domain can act as a dominant negative, dimerizing with full-length TrkC and preventing its activation [47]. Overall, no differences were observed in the full-length TrkC/truncated TrkC ratio, nor in the density of truncated TrkC in the amygdala (Supplementary Fig. S4F, G), PFC (Supplementary Fig. S4G, H) or hippocampus (Supplementary Fig. S4M, N).

#### Amygdala NT3-TrkC signaling rescues fear extinction deficits

**Fig. 5:**
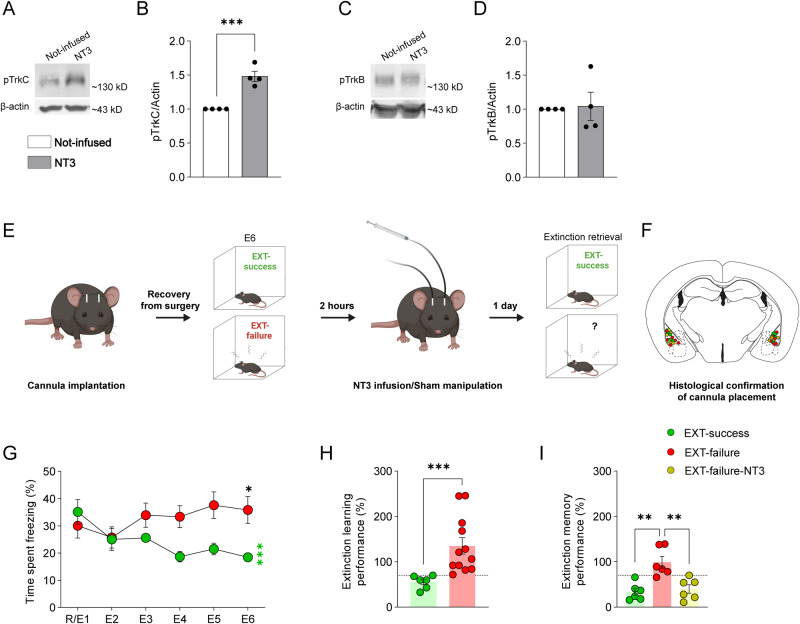
NT3 infusion in the BLA rescues fear extinction deficits in EXT-failure mice. Representative images of western blots for (**A**) pTrkC and (**C**) pTrkB performed using protein extracts from NT3-infused amygdalae *versus* contralateral not-infused amygdalae (*n* = 4 per condition). Quantification of total (**B**) pTrkC and (**D**) pTrkB levels. β-actin was used as a loading control. Two-tailed Student’s *t* test. **E** Schematic representation of experimental conditions and treatments. **F** Schematic representation of cannulas placement in the BLA. Circles represent the tip of the internal cannula. Mice with misplaced cannulas were excluded from the analysis (EXT-success *n* = 2, EXT-failure *n* = 1). **G** Quantification of the percentage of time spent freezing during extinction acquisition session. Fear-conditioned mice were categorized as EXT-success (*n* = 6) or EXT-failure (*n* = 12), according to their extinction learning performance. Repeated measures two-way ANOVA with Tukey multiple comparisons test. * EXT-success, R/E1 vs. E6. **H** Extinction learning performance, expressed as the percentage of freezing levels in E6 relative to freezing in R/E1. Mann–Whitney U test. **I** Extinction memory performance, expressed as the percentage of freezing levels in extinction memory retrieval relative to freezing in R/E1. The dotted line marks the threshold of 30% reduction from R/E1 used to categorize mice as EXT-success or EXT-failure. One-way ANOVA with Tukey multiple comparisons test. E1-E6, extinction trial 1 to 6; ELP, extinction learning performance; EMP, extinction memory performance; NT3, neurotrophin 3; pTrkC, phosphorylated tropomyosin receptor kinase C; pTrkB, phosphorylated tropomyosin receptor kinase B; R/E1, fear retrieval/extinction trial 1. *p ≤ 0.05, **p ≤ 0.01, ***p ≤ 0.001.

Next, we tested whether there is a causal link between TrkC activation in the amygdala and fear extinction performance. To this end, mice were bilaterally implanted with cannulas positioned above the BLA and trained in the CFC and EXT paradigm. At the extinction consolidation window, EXT-failure mice were infused with NT3 and tested for extinction memory (Fig. 5E).

To confirm that NT3 infusion at a dose of 1 µg/µL selectively activates TrkC, NT3 was infused unilaterally (contralateral side was sham-manipulated) in a first batch of unconditioned animals (*n* = 4) and amygdalae were collected 15 min later. Western blot analysis showed an increase in the levels of pTrkC in the NT3-infused amygdalae as compared to the contralateral not-infused amygdalae (Fig. 5A, B, *t* = 6.486, *p* < 0.001). Considering that NT3 can also bind to TrkB receptors, even though with low affinity [48], we measured TrkB activation levels. We did not observe alterations in the levels of pTrkB in the NT3-infused versus contralateral not-infused amygdalae (Fig. 5C, D, *t* = 0. 07351, *p* = 0.9887). Taken together, data confirm that at a dose of 1 µg/µL NT3 infusion in the BLA selectively activates TrkC receptor.

Next, a second batch of cannula-implanted mice was fear conditioned and trained in the EXT paradigm, and categorized as EXT-success or EXT-failure, according to their extinction learning performance. Throughout extinction acquisition trials, EXT-success, but not EXT-failure mice, showed a reduction in their freezing levels (Fig. 5G, extinction trial x group interaction *F*_(5, 80)_ = 7021, *p* < 0.001, R/E1 vs. E6, EXT-success *t* = 4.185, *p* < 0.001; EXT-failure *t* = 2.052, *p* = 0.1989). In particular, in R/E1 trial both EXT-success and EXT-failure mice showed similar robust levels of freezing (EXT-success vs. EXT-failure, *t* = 0.7927, *p* = 0.9696), showing the proper formation of a contextual fear memory. By E6, EXT-success mice showed an average 44% reduction in freezing levels relative to the levels shown in R/E1, as opposed to average 35% increase in freezing levels by EXT-failure mice (Fig. 5H, U = 0, *p* < 0.001).

Two hours after E6, coinciding with the extinction consolidation window, a subset of EXT-failure animals was bilaterally infused with NT3 into the BLA. EXT-failure not-infused and EXT-success animals were sham manipulated. Importantly, when tested for extinction retrieval 24 h later, EXT-failure NT3-infused animals showed an improved extinction memory performance (60% reduction in freezing levels) as compared to EXT-failure sham animals (1% reduction in freezing levels), and comparable to that of EXT-success animals (67% reduction in freezing levels) (Fig. 5I, F_(2, 15)_ = 12.47, *p* < 0.001; EXT-success vs. EXT-failure *q* = 6.404, *p* = 0.001, EXT-success vs. EXT-failure-NT3 *q* = 0.6208, *p* = 0.900, EXT-failure vs. EXT-failure-NT3 *q* = 5.783, *p* = 0.003; *n* = 6 per group).

This set of experiments shows that activation of the NT3-TrkC pathway in the BLA during extinction consolidation is sufficient to rescue fear extinction deficits.

#### NT3-TrkC signaling modulates LTP strength in a GluN2B-dependent mechanism

**Fig. 6:**
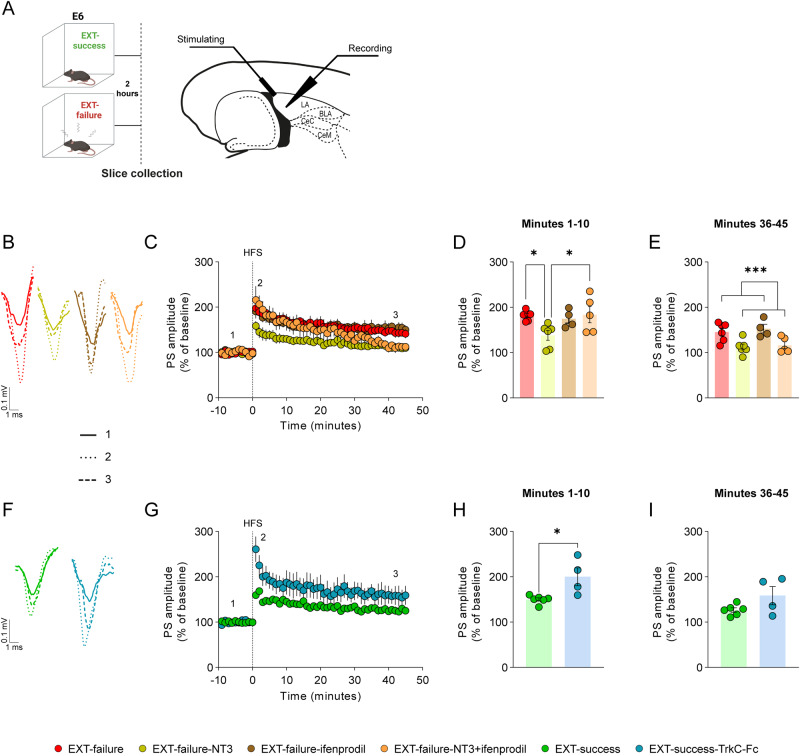
NT3 attenuates LTP at LA synapses in a GluN2B-dependent mechanism. **A** Schematic representation of experimental conditions and localization of stimulating and recording electrode in horizontal brain slices containing the LA. A HFS protocol (three 1 s duration trains of 100 Hz pulses, with 5 s inter-train interval; dashed vertical line) was used to induce ex-vivo LTP in the LA of (**B**–**E**) EXT-failure slices untreated (*n* = 6), treated with NT3 (*n* = 6, 50 ng/mL), treated with ifenprodil (*n* = 4, 3.25 µg/mL), treated with NT3+ifenprodil (*n* = 5, NT3 50 ng/mL and ifenprodil 3.25 µg/mL) and of (**F**–**I**) EXT-success slices untreated (*n* = 6) and treated with TrkC-Fc chimera (*n* = 4, 0.5 µg/mL). Tested drugs were added 45 min before HFS and were maintained in the medium until the end of the experiment. **B** Representative traces for EXT-failure slices untreated and treated with NT3, ifenprodil and NT3+ifenprodil at baseline (^__^), upon 10 min (^…^) and 45 min (---) of HFS. **C** LTP time course recorded for 45 min following induction. Amplitude of PS was averaged for the (**D**) first 10 min and (**E**) last 10 min of recordings following LTP induction. Repeated measures two-way ANOVA with Tukey multiple comparisons test. **F** Representative traces for EXT-success slices untreated and treated with TrkC-Fc at baseline (^__^), upon 10 min (^…^) and 45 min (---) of HFS. **G** LTP time course recorded for 45 min following induction. PS amplitude was averaged for the (**H**) first 10 min and (**I**) last 10 min of recordings following LTP induction. Two-tailed Student’s *t* test. BLA basolateral amygdala, Ce central amygdala, E6 extinction trial 6, HFS high frequency stimulation, LA lateral amygdala, NT3 neurotrophin 3, PS population spikes. TrkC-Fc tropomyosin receptor kinase C–Fc chimera. *p ≤ 0.05.

Finally, we sought to investigate the cellular and molecular mechanisms underlying amygdalar NT3-induced rescue of fear extinction deficits. Here, fear conditioned mice trained in the EXT paradigm were categorized as EXT-success or EXT-failure, according to extinction learning performance (Supplementary Fig. S5A, B). After E6, at extinction consolidation window, mice were killed to prepare horizontal brain slices including the amygdala and ex vivo electrophysiological recordings were performed (Fig. 6A).

First, recapitulating in vivo experiments, we treated EXT-failure slices with NT3 to test whether neurotrophin could also decrease LA LTP (Fig. 6B–E). In the first 10 min following HFS, EXT-failure slices treated with NT3 showed lower potentiation than untreated EXT-failure slices (Fig. 6D; EXT-failure vs. EXT-failure-NT3 *q* = 4.101, *p* = 0.0447; EXT-failure *n* = 6 slices from 6 mice; EXT-failure-NT3 *n* = 6 slices from 3 mice), an effect that was maintained in the last 10 min of recordings (Fig. 6E; NT3 effect *F*_(1, 17)_ = 18.59, *p* = 0.0005).

We hypothesized that the effects of NT3 on LA LTP could be mediated by GluN2B-containing NMDAR. LTP recordings were performed in the presence of NT3 and ifenprodil, an antagonist of GluN2B-containing NMDAR. We observed that in the first 10 min following HFS, ifenprodil administration prevented the effects of NT3 on LTP (Fig. 6D; NT3 x ifenprodil interaction *F*_(1, 17)_ = 5.171, *p* = 0.0362; EXT-failure vs. EXT-failure-NT3+ifenprodil, *q* = 0.1917, *p* = 0.9991, EXT-failure-NT3 vs. EXT-failure-NT3+ifenprodil, *q* = 4.101, *p* = 0.0447; EXT-failure-NT3+ifenprodil *n* = 5 slices from 4 mice). However, in the last 10 min of recordings ifenprodil was no longer able to prevent the effects of NT3 (Fig. 6E; NT3 x ifenprodil interaction *F*_(1, 17)_ = 0.04139, *p* = 0.8412), fitting with the attributed role of NMDAR in the induction phase of LTP [46]. Despite blocking the effects of NT3 on LTP induction, when administered alone ifenprodil did not show an effect neither in the first 10 min after HFS (Fig. 6D; EXT-failure vs. EXT-failure-ifenprodil, *q* = 0.5621, *p* = 0.9780; EXT-failure-ifenprodil *n* = 4 slices from 3 mice) nor in the last 10 min of recordings (Fig. 6E; ifenprodil effect *F*_(1, 17)_ = 0.4615, *p* = 0.5061), demonstrating that GluN2B-containing NMDAR inhibition selectively affects NT3 effects on LA LTP.

Finally, we treated EXT-success slices with TrkC-Fc chimera to scavenge endogenous NT3 and in this way block NT3 signaling (Fig. 6F–I). In the first 10 min following HFS, EXT-success slices treated with TrkC-Fc showed a stronger potentiation than EXT-success untreated slices (Fig. 6H; *t* = 3.084, *p* = 0.0150; EXT-success *n* = 6 slices from 5 mice, EXT-success-TrkC-Fc *n* = 4 slices from 2 mice). However, the difference in LTP was lost in the last 10 min of recordings (Fig. 6I; *t* = 1.884, *p* = 0.0964).

The observed effects were specific to LTP since none of the drugs affected the basal synaptic transmission, as no differences were observed in the I/O curves before and after treatments with NT3, NT3+ifenprodil or TrkC-Fc (Supplementary Fig. S5A–C).

Taken together, our results show that NT3 leads to an attenuation of LA LTP induction in EXT-failure slices, through a GluN2B-dependent mechanism. Conversely, blocking endogenous NT3 signaling with TrkC-Fc in EXT-success slices potentiated LTP, demonstrating that endogenous NT3 is necessary for LA LTP weakening and thereby fear extinction.

**Fig. 7:**
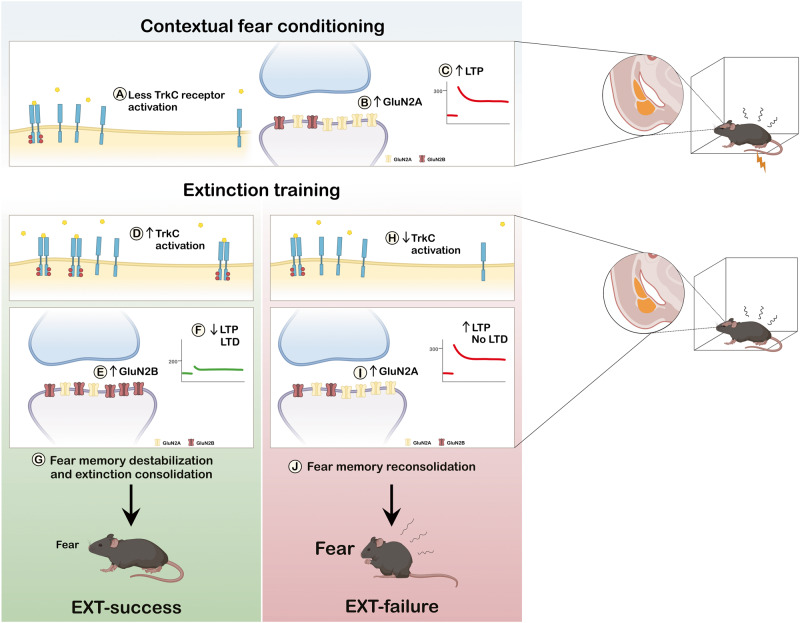
Proposed theoretical model. Contextual fear conditioning induces TrkC inactivation [32] (**A**) and synaptic accumulation of GluN2A-containing NMDAR in the amygdala [26] (**B**), which are responsible for LA LTP [25]. This leads to the induction of GluN2A-dependent LTP in the LA fear-specific microcircuit (**C**), a necessary step for the acquisition of conditioned fear [42]. Given that EXT-success and EXT-failure mice form equally strong fear memories, accumulation of GluN2A-containing NMDAR in synapses and LTP in the LA are not expected to differ in the two groups. Retrieval and concomitant extinction training initiates two parallel processes rendering fear memory labile and amenable for destabilization or reconsolidation on one side and activating the extinction microcircuit on the other side [28, 49]. At this critical timepoint, amygdalar TrkC activation, as observed in EXT-success mice (**D**), will promote the synaptic accumulation of GluN2B- in detriment of GluN2A-containing NMDAR (**E**), resulting in the attenuation of the fear microcircuit, weakening LA LTP (**F**), and activation of the extinction circuit inducing LTD, thereby promoting fear memory destabilization and extinction consolidation (**G**). In EXT-failure animals, insufficient amygdalar TrkC activation (**H**) will prevent the GluN2A to GluN2B switch at synapses (**I**) and thereby promoting the GluN2A-dependent memory stabilization and reconsolidation (**J**), maintaining levels of fear high.

Inter-individual variation in fear extinction relies on a win or lose competition between the fear and extinction microcircuits [49]. Our current findings support a theoretical model where NT3-TrkC system is a key player in mediating this balance (Fig. 7).

## Discussion

Inter-individual differences in the ability to extinguish fear have a dual outcome: first on setting the vulnerability to develop anxiety and fear-related disorders, and second on determining the effectiveness of exposure therapy towards patients in this group of disorders. Indeed, fear extinction mechanisms that support exposure therapy principles [8] are often impaired in patients with fear-related disorders [50]. Here, taking advantage of the individual variation in fear extinction performance, we characterized (at the behavioral, cellular, and molecular level) a model in rodents to study intrinsic group differences in contextual fear extinction. Moreover, we found that NT3-TrkC signaling in the amygdala is sufficient to rescue fear extinction deficits and the underlying synaptic plasticity, in a GluN2B-dependent mechanism.

The behavioral model herein proposed represents a powerful tool to study group differences in fear extinction. Using this model, 40% of fear-conditioned mice were able to extinguish fear (EXT-success) when trained in a contextual extinction paradigm, as opposed to EXT-failure who failed to show a reduction, and in some instances even showed an increase, in freezing levels. Both EXT-success and EXT-failure mice acquire and form equally strong fear memories, suggesting that the observed behavioral differences are specific to the extinction process. Importantly, behavioral differences in EXT-success and EXT-failure mice were maintained 24 h after extinction learning, when mice were tested for extinction retrieval, highlighting the predictive value of learning performance to extinction outcome.

Group differences in fear extinction have been studied before, particularly using models of stress-enhanced fear learning [51–53]. In these models, a prior ‘traumatic’ event results in the enhancement of fear learning and deficits in fear mitigation upon extinction [54]. A modified version of such models to study fear extinction differences in stress-resistant and stress-susceptible mice was previously reported, with the latter showing fear extinction deficits [53]. In these stress-enhanced fear learning models, the different response of the animals to a traumatic event sets the basis for the different extinction performances. Instead, in our model, animals with equally strong fear memories show individual differences in extinction that are inherent to the animals and observed in the absence of any genetic or environmental manipulation.

A model of approach/avoidance conflict task, more closely related to our model, was established to assess individual differences in response to conflicting stimuli [55]. The authors found that animals categorized a priori as ‘avoiding’, in comparison to ‘balancing’, did not extinguish fear [55, 56]. Importantly, behavioral differences were supported by morphological, functional and transcriptomic signatures in the mPFC and amygdala pyramidal neurons, that predispose to maladaptive fear extinction [56].

The formation of fear memories and their extinction is dependent on synaptic plasticity events occurring at amygdalar fear and extinction microcircuits [42]. In particular, the formation of a fear memory is based on the induction of LTP in the thalamic-LA [43] and hippocampal-LA synapses [9]. In turn, the formation of an extinction memory requires the depotentiation of the same synapses potentiated during fear conditioning [24] together with LTD in the LA [22]. In line with the literature and the behavioral data, we observed that EXT-success mice showed a robust LTD and weaker LTP in the LA, while EXT-failure mice showed stronger LTP induction and no LTD. EXT-success and EXT-failure slices could not be distinguished based on the I/O profiles demonstrating that differences in LA synaptic plasticity are not due to altered basal excitability of LA synapses.

Several lines of evidence identified a differential role for GluN2A- and GluN2B-containing NMDAR in the BLA, in fear conditioning and extinction [25–27]. Amygdalar GluN2A-containing NMDAR are required for LTP and for the expression of conditioned fear, while GluN2B-containing NMDAR are required for LTD and fear extinction [25]. Likewise, GluN2A and GluN2B play dissociable roles in the processes of fear memory stabilization and destabilization in the BLA. During fear memory retrieval, rendering fear memories labile [49], GluN2A-containing NMDAR promote stabilization and thereby reconsolidation of fear memories, while GluN2B-containing NMDAR promote their destabilization [26, 27].

Here, we hypothesized that differences in behavior and LA synaptic plasticity observed between EXT-success and EXT-failure mice could be associated with a distinct composition of NMDAR on the surface of amygdala synapses. Indeed, we observed higher levels of GluN2B-containing NMDAR on the surface of EXT-success amygdala synapses, while higher levels of GluN2A were observed in EXT-failure mice. The results are in line with the differential role of the two NMDAR subunits in fear memories, and suggest that, in EXT-success mice, the high levels of GluN2B-containing NMDAR may lead to activation of the extinction microcircuit and to destabilization of the fear memory through synaptic depotentiation of the fear microcircuit. These alterations may allow the extinction microcircuit to prevail over the fear microcircuit. In EXT-failure mice, the higher levels of synaptic GluN2A prevent the depotentiation of LA synapses, driving the stabilization and reconsolidation of the fear memory, which could underlie the increase in freezing levels observed in some EXT-failure mice (extinction learning performance > 100%).

Recently, we observed that formation of a contextual fear memory in mice is associated with downregulation of TrkC activation in the amygdala, hippocampus and prefrontal cortex, key areas of the brain fear network [32]. Here, we measured TrkC activation and density in the above-mentioned brain regions of EXT-success and EXT-failure mice, during extinction consolidation. We observed a higher TrkC activation in the amygdala of EXT-success, as compared to EXT-failure mice. Given the role of neurotrophins as regulators of synaptic plasticity [38], we hypothesized that a differential activation of amygdalar TrkC could account for the differences in synaptic plasticity and the accompanied behavioral performance of EXT-success and EXT-failure mice. Indeed, NT3 infusion rescued fear extinction deficits in EXT-failure mice, demonstrating that TrkC activation in the BLA is sufficient for the proper expression of extinction. Even if at a non-physiological concentration, NT3 infusion in the BLA rescues fear extinction deficits by selectively engaging TrkC receptors, providing a valuable strategy to first unravel the role of this modulatory system in fear extinction. Although NT3 can bind TrkB with low affinity [48], at the dose used in this study, NT3 infusion into the BLA did not trigger TrkB activation. Nevertheless, to understand the physiological role of NT3 in the processing of fear, the effects of NT3 should be confirmed in experiments with a titrated and spatio/temporal-controlled expression of NT3, which still remains to be optimized.

Whether extinction training is required for amygdalar NT3 effects on extinction is still an open question. One possibility is that NT3-TrkC signaling is acting on the extinction network. As an adjuvant, NT3 may promote extinction-specific cellular, molecular and functional changes, triggered by the extinction training process. Alternatively, NT3 may by itself mimic extinction training once the extinction network is recruited by a first unreinforced exposure to the CS. Finally, we cannot exclude that NT3-TrkC signaling may act by depotentiating the amygdalar fear network, leading to destabilization of the fear memory, which per se is an extinction-independent event. Though, a combination of mechanisms cannot be overlooked.

In the amygdala, TrkC is widely distributed across all nuclei [57], suggesting a role for the NT3-TrkC system in this region. However, *Ntf*-3 mRNA expression (which encodes NT3) was not detected in the amygdala [57], suggesting that the action of the NT3-TrkC system in this region may relay on the anterograde transport of NT3 between interconnected brain regions. In the hippocampus, strong levels of *Ntf3* mRNA are detected in hippocampal sub-regions CA2 and CA1, making this the best candidate of NT3 source into the amygdala. Yet another possibility is that very low levels of NT3 are necessary in the amygdala to ensure specific activation of TrkC, as recently reported [48]. However, a limitation imposed by the detection limits of the techniques used cannot be excluded and the expression of *Ntf3* should be re-evaluated in future experiments using new state-of-the-art techniques such as RNA scope.

Next, we questioned which cellular and molecular mechanisms mediate the effects of NT3-TrkC on fear extinction. To that end, we repeated LTP experiments in slices from EXT-success and EXT-failure animals in combination with drugs to manipulate TrkC activation. Importantly, activation of TrkC with NT3 superfusion in EXT-failure slices weakened LTP, restoring EXT-success weak LTP values and confirming that NT3-TrkC signaling attenuates LA LTP. Conversely, blocking endogenous NT3-TrkC signaling with TrkC-Fc chimera in EXT-success slices, strengthened LTP induction. These data converge to point to TrkC activation as a key modulator of the strength of LTP in the LA with an impact on extinction-dependent behavior.

EXT-success animals show increased amygdalar TrkC activation and increased surface levels of GluN2B in amygdala synapses, as compared to EXT-failure animals. We postulated that NT3-TrkC effects on LA synaptic plasticity could be mediated by an effect of this neurotrophin system on NMDAR. Indeed, GluN2B subunit has been associated with fear extinction and LA depotentiation [25]. In EXT-failure slices, combining NT3 treatment with ifenprodil, a selective inhibitor of GluN2B-containing NMDAR, prevented the effects of NT3 in the induction of LTP. These results are in line with previous evidence that it is GluN2A-containing NMDAR that contribute predominantly to the induction of LTP [46]. Therefore, the observation that ifenprodil did not have an effect by itself but was able to block NT3 effects on LTP induction, demonstrates that GluN2B-containing NMDAR signaling is selectively required for NT3-TrkC action on synaptic plasticity, placing GluN2B-containing NMDAR downstream of NT3-TrkC. We hypothesize that NT3-TrkC effects on LA LTP involve GluN2B-containing NMDAR in a first phase, and later the engagement of other mechanisms, possibly including AMPAR phosphorylation and trafficking, local protein synthesis, and morphological and structural changes in the synapses [58, 59].

A connection between NT3-TrkC signaling and NMDAR regulation has been previously shown in motoneurons, where NT3 administration facilitates NMDAR transmission [60], and in cultured hippocampal neurons, where NT3 stimulation increased the expression of GluN2A and GluN2B subunits [61]. This is the first study that provides evidence supporting a connection between these two systems in the amygdala. The potential intracellular mechanism linking NT3-TrkC to GluN2B is yet to be investigated. A good candidate is Src kinase, a non-receptor protein tyrosine kinase with a well-known role in intracellular signal transduction [62]. Src can be activated through direct interaction with phosphorylated TrkC, thereby mediating its intracellular effects [63]. The C-terminal domain of GluN2B is a direct target of Src that promotes GluN2B-containing NMDAR activity and trafficking [64]. In addition, it has been shown that Src-dependent phosphorylation promotes the localization of GluN2B-containing NMDAR on the postsynaptic surface of amygdala synapses, a mechanism involved in synaptic plasticity and amygdala-dependent learning [65]. Whether TrkC-dependent activation of Src could mediate the GluN2B-dependent effects of NT3 on LA LTP, or the accumulation of GluN2B-containing NMDAR on the surface amygdala synapses during fear extinction, has yet to be proven.

We here demonstrated that NT3-TrkC signaling rescues fear extinction in EXT-failure animals and the underlying LA synaptic plasticity, in a GluN2B-dependent mechanism. Modulation of amygdalar NMDAR has already been exploited in pharmacological treatments to potentiate fear extinction in animals and humans [66]. Of particular relevance is the case of D-cycloserine – a partial agonist of the NMDAR glycine binding site – which has been successfully tested in combination with exposure therapy, enhancing its efficacy in the treatment of anxiety disorders [67, 68]. Combining psychotherapy with adjuvants, particularly drugs that potentiate biological mechanisms of psychotherapy, i.e. extinction [69, 70], is an attractive option being explored in the field of anxiety and fear-related disorders.

Behavioral differences observed between EXT-success and EXT-failure animals are empiric and cannot be attributed to the administration of stressors, genetic or pharmacological manipulations known to impair extinction [25, 29, 71]. Having a population with naturally occurring different levels of extinction is of relevance to identify molecules of interest for the development of personalized treatments for those patients with extinction deficits. We hypothesize that epigenetics might be playing a major role in setting inter-individual differences in fear extinction in this so said ‘homogenous’ population of mice. Yet research is needed to identify the origin of this variability.

In the present work, based on inter-individual differences in fear extinction, we present an empiric model in rodents to investigate from the behavioral down to the cellular and molecular levels differences between mice that successfully extinguish fear and those that fail. In addition, we identified a key role for the NT3-TrkC system in fear extinction, through modulation of amygdalar NMDAR composition and synaptic plasticity. Our study prompts TrkC as a molecule of interest worthy of further research in the development of drugs to improve the efficacy of exposure therapy. TrkC can be part of a new generation of mechanism-based therapeutic approaches, much in need towards anxiety and fear-related disorders.

## Material and methods

### Animals

Young adult (8 to 12 weeks old) C57BL/6J male mice (Charles River) were housed in groups of four in transparent plastic cages measuring 24.5 × 24.5 × 15 cm. We used *Black poplar/aspen* shavings as litter bedding, two sheets of tissue as nesting material, and an in-house autoclaved cardboard cylinder as enrichment. Food and water were available *ad libitum*. Rooms were maintained under standard environmental conditions (humidity 55 ± 10%; temperature 20–24 °C) with a 12 h light/dark cycle (lights on at 8:00 a.m.). Animals were monitored every day.

### Behavioral tests

A first group of animals was submitted to a behavioral battery starting with the EPM to monitor anxiety-like behavior, followed by the OF to assess spontaneous exploratory activity, and finally the CFC and EXT paradigm to study aversive associative learning and memory. Subsequent batches of animals were submitted only to the CFC and EXT paradigm. Group sizes were estimated based on results from previous publications [29, 32, 37].

### EPM test

The EPM apparatus consisted of two opposite open arms and two opposite closed arms (65 cm arm to arm × 5 cm wide), elevated 50 cm above the floor. Animals were placed in the center of the maze facing a closed arm and allowed to explore the maze for five min, under dim light conditions. The movement of mice was tracked using an automated tracking system (ANY-mazeTM video tracking system, Stoelting Co., Wood Dale, IL, USA). The total distance traveled, the distance traveled and the time spent in each arm of the maze was analyzed. The total distance traveled was used as a measure of locomotor activity and the percentage of distance traveled and the time spent in the open arms was used to assess anxiety-like behavior.

### OF test

The OF consisted of a squared arena (in cm: 38 width × 38 length × 38 height). Animals were placed in one of the corners and allowed to explore the arena in a 10-min session, under dim light conditions. The movement of mice was tracked using an automated tracking system (ANY-mazeTM video tracking system, Stoelting Co.). The total distance traveled was used as a measure of exploratory activity and locomotion, and the percentage of distance traveled and the time spent in a predefined center area *versus* the rest of the arena was used to assess anxiety-like behavior.

### CFC and EXT paradigm

The co-occurrence of an aversive unconditioned stimulus (US, a mild foot-shock) together with a neutral CS (the context itself) generates a conditioned response to the CS in the absence of the US. The learned association is amenable to extinction through successive presentations of the CS in the absence of the US, with the formation of a new CS-no US association.

Mice were trained in the CFC and EXT paradigm, as we described previously [29, 37]. On the first day, for habituation session, mice were placed in the conditioning cage and allowed to freely explore it for 3 min. On the second day, mice were placed in the same cage for fear acquisition session, consisting in a 5-min session including 2 min of free exploration followed by five unconditioned stimuli foot-shocks (US1-US5, 0.5 mA, 2 s) separated by different intervals of time (between 15–45 s). On the third day, fear memory retrieval (R) was assessed, followed by training on fear extinction acquisition. Here, mice were placed in the conditioning cage for six 2-min trials (R/E1-E6), each separated by one-hour interval, in which no foot-shocks were administered. On the fourth day, mice were tested for extinction memory retrieval (ER), consisting in a single 2-min session in which mice were placed in the conditioning cage with no foot-shocks administration.

All sessions were recorded, and freezing levels were analyzed in the 2-min sessions. In the fear acquisition session, freezing levels were analyzed in the 15 s following each shock. A group of mice that did not receive any foot-shock (CTRL-no shock) was included as control group.

Extinction learning performance (ELP) was defined for each individual as the ratio between freezing levels in E6 and those shown in R/E1 $({ELP}=\frac{{freezing\; levels\; E}6}{{freezing\; levels\; R}/E1}x100).$ Likewise, extinction memory performance (EMP) was defined as the ratio between freezing levels in ER and those shown in R/E1 $({EMP}=\frac{{freezing\; levels\; ER}}{{freezing\; levels\; R}/E1}x100).$ We categorized as EXT-success the mice that showed a relative reduction in freezing levels from R/E1 to E6 of at least 30% (ELP ≤ 70%), as opposed to EXT-failure mice who failed to reduce their freezing levels, or even show an increase (ELP > 70%).

All procedures were performed in a fear conditioning system (UgoBasile, Gemonio, Italy) and recorded using the incorporated ANY-maze software for fear conditioning. Freezing behavior was measured manually by two experienced researchers that were blind to the condition and/or treatment.

### Isolation of synaptoneurosomes from amygdalae of fear extinction-trained mice

Synaptoneurosomes were prepared as described before [72], with slight modifications. Mice were trained in the CFC and EXT paradigm and sacrificed two hours after trial E6, corresponding to the extinction memory consolidation window. Amygdalae were dissected and pooled in groups of four (from two animals). The tissue was minced and homogenized with a Kontes Dounce Tissue Grinder, using first a pestle with large clearance, 0.089–0.165 mm (10 strokes), followed by a small clearance pestle, 0.025–0.076 mm (10 strokes), in an isolation buffer containing 0.32 M sucrose, 10 mM HEPES-Tris pH 7.4 and 0.1 mM EGTA pH 8.0, to a final volume of 5 mL. After centrifugation for 3 min at 1,000 *g*, the supernatant was collected and passed initially through three-layer nylon membranes (150 and 50 μm pore size) and finally through an 8 μm pore size filter (Millipore, Burlington, MA, USA). The flow-through was centrifuged for 15 min at 10,000 *g*, and the resulting pellet was resuspended in isolation buffer to a final volume of 1 mL. Resuspended synaptoneurosomes were plated on coverslips for live immunofluorescence. All procedures were done at 4 °C.

### Live immunofluorescence of membrane glutamate receptors in synaptoneurosome preparations

Resuspended synaptoneurosomes were plated on 10 mm diameter coverslips coated with poly-D-lysine (0.1 mg/mL, 50 µL/coverslip) and left in a humid dark chamber for 1 h at room temperature (RT) for synaptoneurosomes to adhere to the coverslip. Live immunofluorescence was performed by incubating synaptoneurosomes for 10 min at RT with the appropriate primary antibody diluted in isolation buffer. The following primary antibodies were used: rabbit anti-GluN2A (extracellular) (1:100, AGC-002, Alomone, Israel), rabbit anti-GluN2B (extracellular) (1:100, AGC-003, Alomone), rabbit anti-GluA1 (extracellular) (1:100, AGC-004, Alomone) and rabbit anti-GluA2 (extracellular) (1:100, AGC-005, Alomone). Next, synaptoneurosomes were fixed with 4% paraformaldehyde (PFA) for 15 min at RT, and permeabilized with phosphate-buffered saline with Triton X100 (PBS-TX) 0.25% for 5 min at RT. The preparation was blocked using 10% bovine serum albumin (BSA) in PBS for 1 h at RT, and incubated with the primary antibodies mouse anti-PSD-95 (1:200, MA1–045, Thermo Fisher Scientific, MA, USA) and guinea pig anti-VGlut1 (1:5000, AB5905, Sigma-Aldrich, MA, USA) diluted in 3% BSA in PBS, overnight at 4 °C. The following day, coverslips were incubated with respective secondary antibodies diluted in 3% BSA in PBS for 1 h at RT: goat anti-rabbit IgG alexa fluor 568 (1:500, A11036, Thermo Fisher Scientific), goat anti-mouse IgG alexa fluor 488 (1:500, Thermo A11001, Fisher Scientific) and goat anti-guinea pig IgG alexa fluor 647 (1:500, A21450, Thermo Fisher Scientific). Coverslips were mounted on microscope slides with mounting medium (S3023, Agilent Dako, CA, USA).

### Glutamate receptors fluorescence imaging acquisition and quantitative analysis in synaptoneurosomes

Fluorescence imaging was performed on a Carl Zeiss Axio Imager Z2 widefield fluorescence microscope. Images were obtained with a Plan-Apochromat 100×1.4 numerical aperture oil objective, equipped with a Zeiss HRm AxioCam. Zeiss filter sets 31, 38 (HE) and 50 were used. Phase-contrast images were also acquired. Identical exposure time and light intensity were used in different conditions of each experiment.

Images were analyzed using ImageJ software (National Institutes of Health, MD, USA) with a specific macro designed *in house* for this experiment. The identification of intact synaptoneurosomes required the fulfillment of the following parameters: juxtaposition of presynaptic (VGluT1) and postsynaptic (PSD95) protein clusters, presence of sealed synaptoneurosomes (visible as dark objects in the phase-contrast image), fit dimension (300–1000 nm) and shape (snowman-like structure) criteria, as described in [73]. Identification of synaptoneurosomes was performed by a researcher blind to the signal of target molecules, i.e. GluN2A, GluN2B, GluA1 and GluA2. Regions of interest (ROIs) were manually drawn around approximately 500–600 synaptoneurosomes per condition, and background and thresholds were set for GluN2A, GluN2B, GluA1 and GluA2 signal. The percentage of GluN2A-, GluN2B-, GluA1- and GluA2-positive synaptoneurosomes, and the integrated density of GluN2A, GluN2B, GluA1 and GluA2 staining in synaptoneurosomes, were quantified.

### Tissue collection and total protein extraction

Mice were killed at the end of the extinction acquisition session, 2 h after trial E6, corresponding to the extinction memory consolidation window. Amygdalae, hippocampi and PFC were dissected and immediately frozen at −80 °C. PFC dissection included approximately 2.5 mm of the most frontal part of the brain with coordinates from Paxinos & Franklin mouse brain atlas [74]: anterior-posterior +3.3 to +1.8, excluding the portion of the olfactory bulbs, olfactory areas and nuclei. To dissect the amygdala, the brain was positioned upside down and 2 vertical and 2 horizontal parallel cuts were done around the hypothalamus. Next, bilateral incisions on the edge of the cortex were performed and the regions inside the different cuts, corresponding to the amygdalae, were carefully lifted and collected.

Amygdalae, hippocampi and PFC were homogenized respectively in 100 μL, 300 μL and 200 μL of ice-cold radioimmunoprecipitation assay (RIPA) buffer (150 mM NaCl, 50 mM Tris-HCl pH7.4, 5 mM EGTA, 1% Triton X-100, 0.5% deoxycholate and 0.1% SDS, pH 7.5), supplemented with cocktail inhibitors of proteases (cOmplete protease inhibitor cocktail, Roche, Switzerland) and phosphatases (PhosSTOP, Sigma-Aldrich). Lysates were placed in an orbital rotator for 30 min at 4 °C and then centrifuged at 16,000 *g* for 30 min at 4 °C. The supernatant was collected and stored at −80 °C. The total protein concentration was measured using the bicinchoninic acid (BCA) protein assay kit (Sigma-Aldrich).

### Western blot

Thirty μg of total protein extracts, denatured for 30 min at RT, were resolved by electrophoresis in 7% sodium dodecyl sulfate-polyacrylamide gels (SDS-PAGE) and transferred to polyvinylidene fluoride (PVDF) membranes (Immobilon-P, Merck Millipore, MA, USA) for 1 h at 100 V, at 4 °C. Membranes were then blocked for 1 h at RT in Tris buffered saline – tween-20 (TBS-T: 137 mM NaCl, 20 mM Tris-HCl, pH 7.6 with 0.1% tween-20) containing 5% (w/v) low fat milk and incubated with primary antibodies diluted in blocking solution, overnight at 4 °C. Primary antibodies were: rabbit anti-phospho-TrkC Tyr516 (1:500 dilution, PA5–39755, Thermo Fisher Scientific); rabbit anti-TrkC (1:1000, #3376, Cell Signaling Technology, MA, USA); mouse anti-β-actin antibody (1:5000, A5441, Sigma-Aldrich); rabbit anti-phospho-TrkB Tyr816 (1:500, ABN1381, Sigma-Aldrich). Membranes were then incubated with the appropriate secondary antibodies: alkaline phosphatase-conjugated donkey anti-rabbit (1:10000, A16026, Thermo Fisher Scientific) or alkaline phosphatase-conjugated donkey anti-mouse (1:10000, A16014, Thermo Fisher Scientific), diluted in 0.5% milk/TBS-T for one hour at RT. Finally, membranes were incubated with ECF substrate (GE Healthcare, IL, USA), and images were acquired with the ChemiDoc Imaging System (Bio-Rad, CA, USA). When needed, membranes were stripped with 0.2 M NaOH for 20 min at RT, blocked again with 5% milk/TBS-T and re-probed for other proteins of interest. β-actin was used as a loading control. Bands were quantified using ImageJ software (National Institutes of Health) following the guidelines of Gassmann and colleagues [75].

### Stereotaxic surgery

Eight-week-old C57BL/6J mice were bilaterally implanted with guide cannulas positioned above the BLA. Animals were anesthetized with a mixture of medetomidine (1 mg/kg, i.p.) and ketamine (75 mg/kg, i.p.). After complete loss of reflexes, the head was fixed in a stereotaxic apparatus (Stoelting Co.) and, following mouse brain atlas coordinates [74], two holes were opened in the skull, and guide cannulas (outer diameter 0.5 mm, inner diameter 0.25 mm, AISI 304, Unimed S.A., Switzerland) were implanted bilaterally 1 mm above the target region, corresponding to the BLA, as follows: anteroposterior, −1.6 mm; mediolateral, ±3.3 mm; dorsoventral, −4 mm. Guide cannulas were fixed with dental cement (DENTALON® plus, Heraeus Kulzer, Germany). At the end of the surgical procedure, the skin was closed with tissue glue (Medbond, CP Medical), anesthesia was reversed with atipamezole (2 mg/kg, i.p.) and analgesia was provided by buprenorphine injection (0.05 mg/kg, s.c.). In the 72 h post-op period, mice received meloxicam every 24 h (2 mg/kg, s.c.).

### Intra-BLA NT3 administration

Recombinant human NT3 (N-260, Alomone) was locally infused through pre-implanted guide cannulas. Infusions were performed using an internal cannula (outer diameter 0.2 mm, inner diameter 0.09 mm, AISI 316 L, Unimed S.A.) connected to a 10 μL syringe (1701 RN, Hamilton, NV, USA) through a 15 cm long PVC tube (outer diameter 0.64 mm, inner diameter 0.28 mm, 51150, Stoelting Co.). Mice were manually immobilized, the internal cannula was inserted into the guide cannula, exiting into the brain for 1 mm, and the infusion was performed at a rate of 750 nL/min, using a Quintessential Stereotaxic Injector (Stoelting Co.). The internal cannula was gently removed 1 min after the infusion to allow drug diffusion and avoid reflux.

NT3 (0.75 µL, 1 µg/µL) was infused in mice trained in the CFC and EXT paradigm. Freezing levels were analyzed immediately after performance in each trial (E1 to E6). By E6, extinction learning performance was calculated and mice were categorized in EXT-success or EXT-failure. Two hours after the last trial of extinction acquisition (E6), EXT-failure animals, randomly assigned, received either bilateral infusion of NT3 or sham manipulation (immobilization, insertion of the internal cannula). EXT-success animals were also sham manipulated. The following day, all animals were tested for extinction memory retrieval. Mice were killed and brains processed for histology to confirm cannula implantation site.

An independent group of animals with bilateral implanted cannulas received NT3 (0.75 µL, 1 µg/µL) unilaterally. Mice were killed 15 min after infusion, amygdalae were dissected and total protein extracts prepared, as described above. TrkC and TrkB activation (measured by their phosphorylation levels) in NT3 infused and contralateral not-infused amygdalae were measured by western blot.

### Histological confirmation of cannula implantation sites

Mice were intracardially perfused with 0.1 M PBS to clean off excess blood, followed by 4% paraformaldehyde (PFA) solution in PBS to fix brain tissue. Brains were extracted, kept in 4% PFA solution in PBS for 24 h and then transferred to a 30% sucrose solution until brains sank. Brains were then frozen in optimal cutting temperature compound (OCT, 361603E, VWR chemicals, PA, USA).

Forty-μm coronal slices were obtained in a cryostat (CryoStar NX50, Thermo Fisher Scientific), mounted on gelatin coated slides and counter-colored with Nissl staining. Briefly, after rehydration slices were immersed in 0.5% cresyl violet solution for 4 min. Then, slices were washed, dehydrated and mounted in Eukitt (03989, Sigma-Aldrich).

Brightfield imaging was performed on a Carl Zeiss Axio Imager Z2 widefield microscope. Images were obtained with EC Plan Neofluar 5×0.16 numerical aperture air objective, equipped with a Zeiss HRc Axiocam. The site of the tip of internal cannulas was assessed by overlapping the images with the corresponding slice from Paxinos & Franklin mouse brain atlas [74]. Image dimensions were adjusted to fit in the model slices, and the tip of the internal cannula was marked. Mice with misplaced cannulas were excluded from the analysis (EXT-success *n* = 2, EXT-failure *n* = 1).

### Ex-vivo electrophysiology recordings

Mice were trained in the CFC and EXT paradigm and killed two hours after E6 trial, during extinction consolidation window. The brains were extracted and kept in ice-cold artificial cerebrospinal fluid (aCSF: NaCl 124 mM, KCl 3 mM, NaH_2_PO_4_ 1.25 mM, NaHCO_3_ 26 mM, MgSO_4_ 1 mM, CaCl_2_ 2 mM, glucose 10 mM, and gassed with a 95% O_2_ 5% CO_2_ mixture). Lateral amygdalae-containing 400 µm horizontal slices were cut with a Leica Vibratome (Leica, Wetzlar, Germany) and left to recover in a holding chamber with oxigenated aCSF at 32–34 °C for at least 1 h.

Recordings were performed by submerging each slice in a 1 mL recording chamber with continuous superfusion of oxygenated aCSF at 30.8 °C, at a flow rate of 3 mL/min. A stimulating bipolar concentric electrode was placed inside the LA, in the proximity of the external capsule. Population spikes (PS) were recorded every 20 s, with a heat-pulled borosilicate glass recording electrode filled with 4 M NaCl (2–5 MΩ resistance) placed within the lateral amygdala. The stimulation was performed using either a Grass S44 or a Grass S48 square pulse stimulator (Grass Technologies, Carlow, Ireland) or a Digitimer DS3 stimulator (Digitimer LTD, Welwyn Garden City, UK), with rectangular pulses of 0.1 millisecond applied every 20 s. After amplification (ISO-80, World Precision Instruments, Hitchin, UK), the recordings were digitized (BNC-2110, National Instruments, Austin, TX, USA), averaged in groups of 3 and analyzed using WinLTP software [76]. After I/O curves were obtained, slices were continuously superfused with aCSF, aCSF + NT3 (50 ng/mL, N-260, Alomone), aCSF + NT3 (50 ng/mL) + ifenprodil (3.25 µg/mL, I2892, Sigma-Aldrich), aCSF + ifenprodil (3.25 µg/mL) or aCSF + TrkC-Fc (0.5 µg/mL, RPC-004, Alomone), at 30.8 °C, for 30 min. A second I/O curve was obtained to determine the maximal response, and a baseline was recorded for 10 min with a stimulation eliciting 40% of maximal PS amplitude for LTP and 60% for LTD experiments. LTP was induced with three trains of 100 Hz pulses (1 s duration) delivered every 5 s, and PS was recorded for 45 min following LTP induction. LTD was induced by delivering 900 stimuli at 1 Hz and PS recorded for 45 min following LTD induction. In slices treated with NT3, NT3 + ifenprodil, ifenprodil and TrkC-Fc, drugs were kept circulating for the total duration of the experiment.

For LTP and LTD analysis, the amplitude of the PS was normalized to the average of the baseline and expressed as percentage of baseline response. For each slice, we calculated the average amplitude of the first 10 min of recordings (min 1–10) and the last 10 min of recordings (min 36–45) after LTP or LTD induction, and compared between groups.

### Statistical analysis

All data were analyzed using GraphPad Prism software (Version 8.4.3, GraphPad Software, CA, USA). The normality of the data was assessed using the Shapiro-Wilk test. In case of normal distributions, outliers were identified by the ROUT method (Q = 1%) and removed. In the case of significant difference of variances, Welch correction was applied for Student’s *t* test and Brown–Forsythe for ANOVA. Behavioral data from fear acquisition and extinction acquisition were analyzed using repeated measures two-way ANOVA with *post hoc* Tukey or Sidak test, for between groups and within groups comparisons, respectively. Data from fear retrieval, extinction learning performance and extinction memory performance, and western blots, were analyzed with one-way ANOVA followed by Tukey’s multiple comparisons test in the case of normal distributions or Kruskal-Wallis test followed by Dunn’s multiple comparisons test in case of non-normal distributions. Data from synaptoneurosomes analysis and part of the electrophysiology experiments were analyzed with the two-tailed Student’s *t* test in the case of normal distributions or with the Mann-Whitney U test in the case of non-normal distributions. The other electrophysiology experiments were analyzed using two-way ANOVA with *post hoc* Tukey or Sidak test. Pearson’s coefficients were calculated to assess correlations between extinction learning and extinction memory and between time spent in the open arms in the EPM and extinction learning. In the case of normal distributions, column graphs represent averages ± standard error of the mean (SEM). In the case of non-normal distributions, box and whiskers graphs were used, with the horizontal line representing the median, the box representing the first and third quartiles, and the whiskers showing minimum and maximum values. Depending on the experimental conditions, data were normalized on the average of CTRL-no shock or EXT-success groups. Statistical significance was set at 0.05 (*p ≤ 0.05, **p ≤ 0.01, ***p ≤ 0.001).

### Supplementary information

Supplementary material

## Data Availability

All data generated in this study, including raw data files, Excel spreadsheets, and GraphPad files, are available upon reasonable request to the corresponding author via email.
