# Supplementary material for: The amygdala NT3-TrkC pathway underlies inter-individual differences in fear extinction and related synaptic plasticity
Source: Mol Psychiatry. 2024 Jan 17;29(5):1322–37. doi: 10.1038/s41380-024-02412-z (PMC11189811; doi:10.1038/s41380-024-02412-z)
Supplement: Supplementary file 1 — Supplementary material [file 41380_2024_2412_MOESM1_ESM.docx]

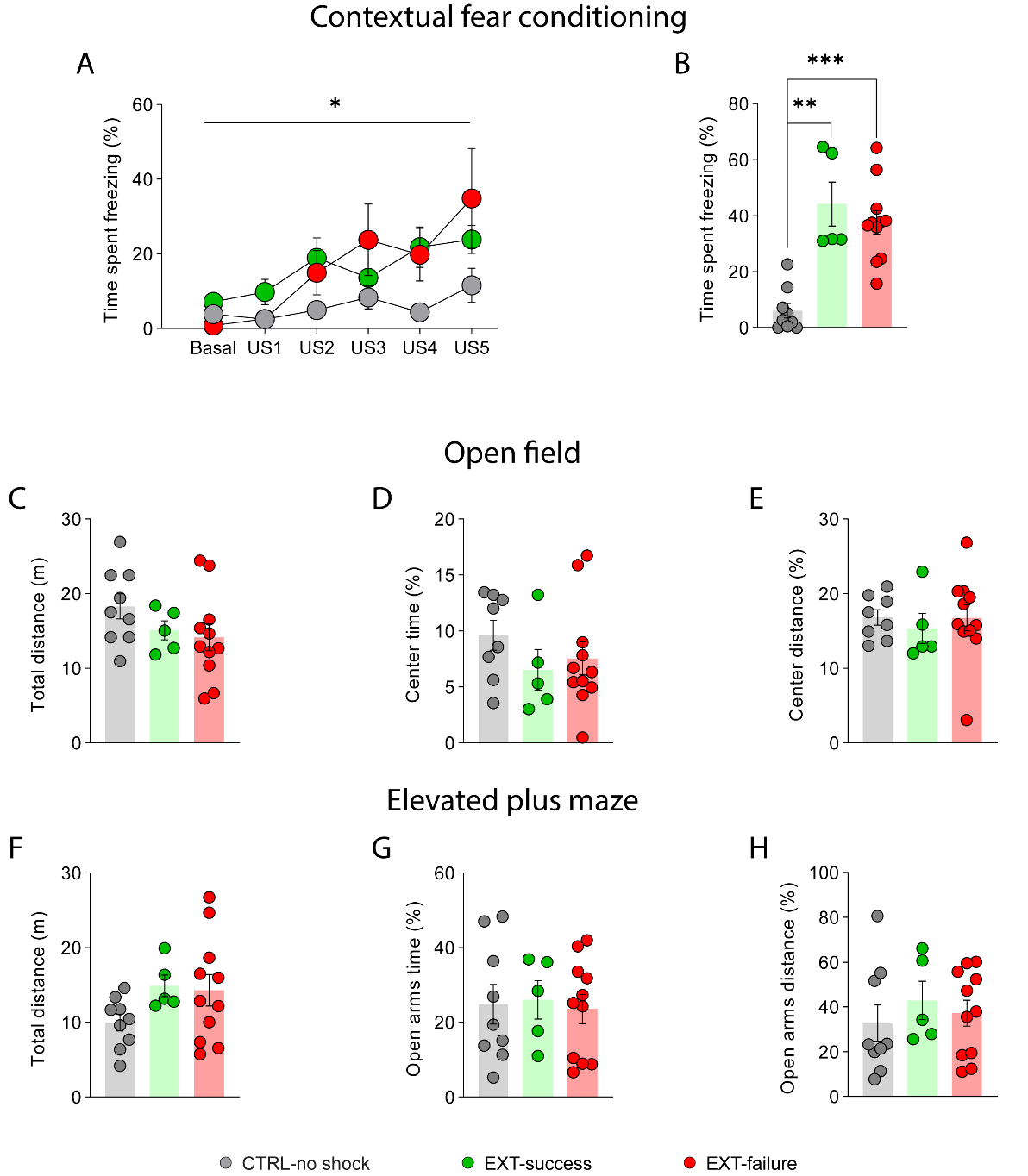


**Fig. S1. EXT-success and EXT-failure mice show similar conditioned fear learning, fear retrieval and basal anxiety-like behavior. (A)** Quantification of the percentage of time spent freezing during contextual fear conditioning. EXT-success (n = 5) and EXT-failure (n = 11) mice progressively increased their freezing levels with consecutive shock administrations, while unconditioned CTRL-no shock mice (n = 9) showed residual levels of freezing (repeated measures two-way ANOVA, US x group interaction F_(10, 110)_ = 2.107, p = 0.0296). **(B)** Quantification of the percentage of time spent freezing during fear memory retrieval. EXT-success and EXT-failure mice showed higher levels of freezing compared to CTRL-no shock group, demonstrating the proper acquisition of conditioned fear (Kruskal-Wallis H = 16.21, p < 0.001; Dunn's multiple comparison test, CTRL-no shock vs. EXT-success Z = 3.140, p = 0.005, CTRL-no shock vs. EXT-failure Z = 3.650, p < 0.001, EXT-success vs. EXT-failure Z = 0.2062, p > 0.999). **(C-E)** Quantification of the total distance travelled **(C)**, percentage of time spent in the center **(D)** and percentage of distance travelled in the center **(E)** in the open field test. No differences were observed among CTRL-no shock, EXT-success, and EXT-failure groups, demonstrating similar levels of basal anxiety-like behavior (total distance, one-way ANOVA, F _(2, 22)_ = 1.662, p = 0.213; center time, one-way ANOVA, F _(2, 21)_ = 0.8838, p = 0.428; center distance, one-way ANOVA, F _(2, 21)_ = 0.1832, p = 0.834). **(F-H)** Quantification of the total distance travelled **(F)**, percentage of time spent in open arms **(G)** and percentage of distance travelled in open arms **(H)** in the elevated plus maze. No differences were observed among CTRL-no shock, EXT-success and EXT-failure groups, demonstrating similar levels of basal anxiety-like behavior (total distance, one-way ANOVA, F _(2, 22)_ = 2.072, p = 0.150; open arms time, one-way ANOVA, F _(2, 22)_ = 0.05706, p = 0.945; open arms distance, one-way ANOVA, F _(2, 22)_ = 0.3766, p = 0.691). US, unconditioned stimulus. *p ≤ 0.05, **p ≤ 0.01, ***p ≤ 0.001.


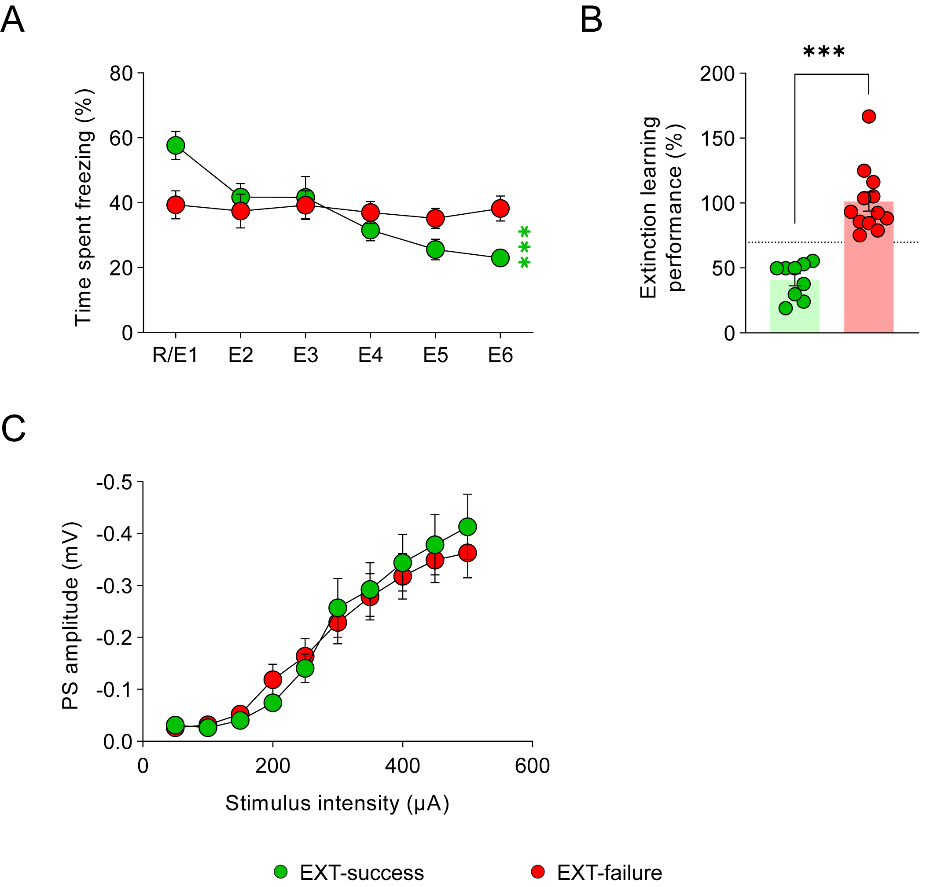


**Fig. S2. Fear extinction performance and I/O curves of fear conditioned mice used in electrophysiology experiments. (A)** Quantification of the percentage of time spent freezing during the extinction acquisition session of fear conditioned mice. EXT-success (n = 9) mice significantly decreased their freezing levels from R/E1 to E6, while EXT-failure (n = 12) kept their freezing levels high (repeated measures two-way ANOVA, extinction trial x group interaction F_(5, 95)_ = 11.20, p < 0.001; Sidak multiple comparisons test, EXT-success, R/E1 vs. E6, t = 8.266, p < 0.001; EXT-failure, R/E1 vs. E6, t = 0.5009, p = 0.9987). Green *: EXT-success, R/E1 vs. E6. **(B)** Quantification of the ELP ratio of fear conditioned mice trained in the extinction paradigm. Mice were categorized as EXT-success or EXT-failure according to their ELP. The dotted line marks the threshold of 30% reduction in freezing, used to categorize mice as EXT-success (Mann-Whitney U test, U = 0, p < 0.001). **(C)** I/O curves of EXT-success (n = 16) and EXT-failure (n = 15) LA-containing horizontal slices. No differences were observed in the I/O profiles (repeated measures two-way ANOVA, stimulus x group interaction F_(9, 261)_ = 0.5127, p = 0.8649), showing that the two groups have a similar LA basal excitability. I/O, input/output; R, fear retrieval; E1 to E6, extinction trials; ELP, extinction learning performance; PS, population spike. ***p ≤ 0.001.


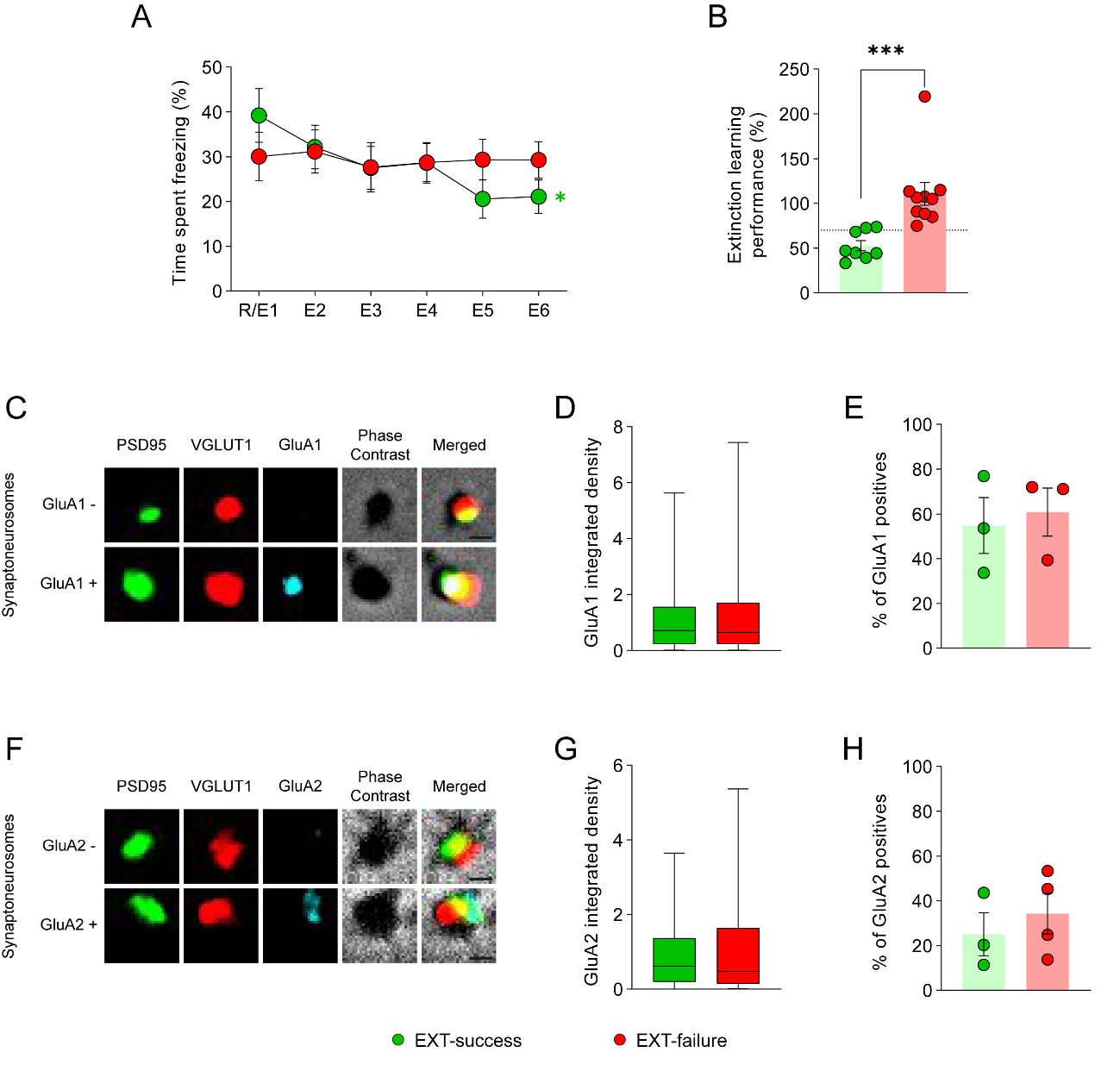


**Fig. S3. Fear extinction performance of conditioned mice used in synaptoneurosome experiments. (A)** Quantification of the percentage of time spent freezing during extinction acquisition of fear conditioned mice. EXT-success (n = 8) mice significantly decreased their freezing levels from R/E1 to E6, while EXT-failure (n = 13) kept their freezing levels high (repeated measures two-way ANOVA, extinction trial x group interaction F_(5, 80)_ = 3.595, p = 0.0055; Sidak multiple comparisons test, EXT-success, R/E1 vs. E6, t = 5.124, p = 0.0202; EXT-failure, R/E1 vs. E6, t = 0.3558, p > 0.9999). Green *: EXT-success, R/E1 vs. E6. **(B)** Quantification of the ELP ratio of fear conditioned mice trained in the extinction paradigm. Mice were categorized as EXT-success or EXT-failure according to their ELP. The dotted line marks the threshold of 30% reduction in freezing, used to categorize mice as EXT-success (Mann-Whitney U test, U = 0, p < 0.001). **(C, F)** Representative images of synaptoneurosomes isolated from the amygdalae of EXT-success and EXT-failure mice, live stained for GluA1 and GluA2 subunits of AMPA receptors. Synaptoneurosomes were identified with co-staining against the postsynaptic marker PSD95 and the presynaptic marker VGlut1 and inspection of intact membranes by phase contrast. **(D, G)** Integrated density of GluA1 and GluA2 signal was quantified in synaptoneurosomes isolated from the amygdalae of EXT success (GluA1 n = 280, GluA2 n = 93) and EXT-failure (GluA1 n = 439, GluA2 n = 173) mice. No differences were observed in synaptic surface expression for GluA1 and GluA2 between EXT-success and EXT-failure mice (GluA1, Mann-Whitney U test, U = 60949, p = 0.8507; GluA2, Mann-Whitney U test, U = 7895, p = 0.8034). **(E, H)** The percentage of GluA1- and GluA2-positive amygdala synaptoneurosomes was calculated for EXT-success (n = 3) and EXT-failure (n = 4) animals. No differences were observed in the percentage of GluA1- and GluA2-positive synaptoneurosomes between EXT-success and EXT-failure mice (GluA1, Mann-Whitney U test, U = 60949, p = 0.8507; GluA2, Student *t*-test, t = 0.6903, p = 0.5207). R, fear retrieval; E1 to E6, extinction trials; ELP, extinction learning performance. *p≤0.05, ***p ≤ 0.001.


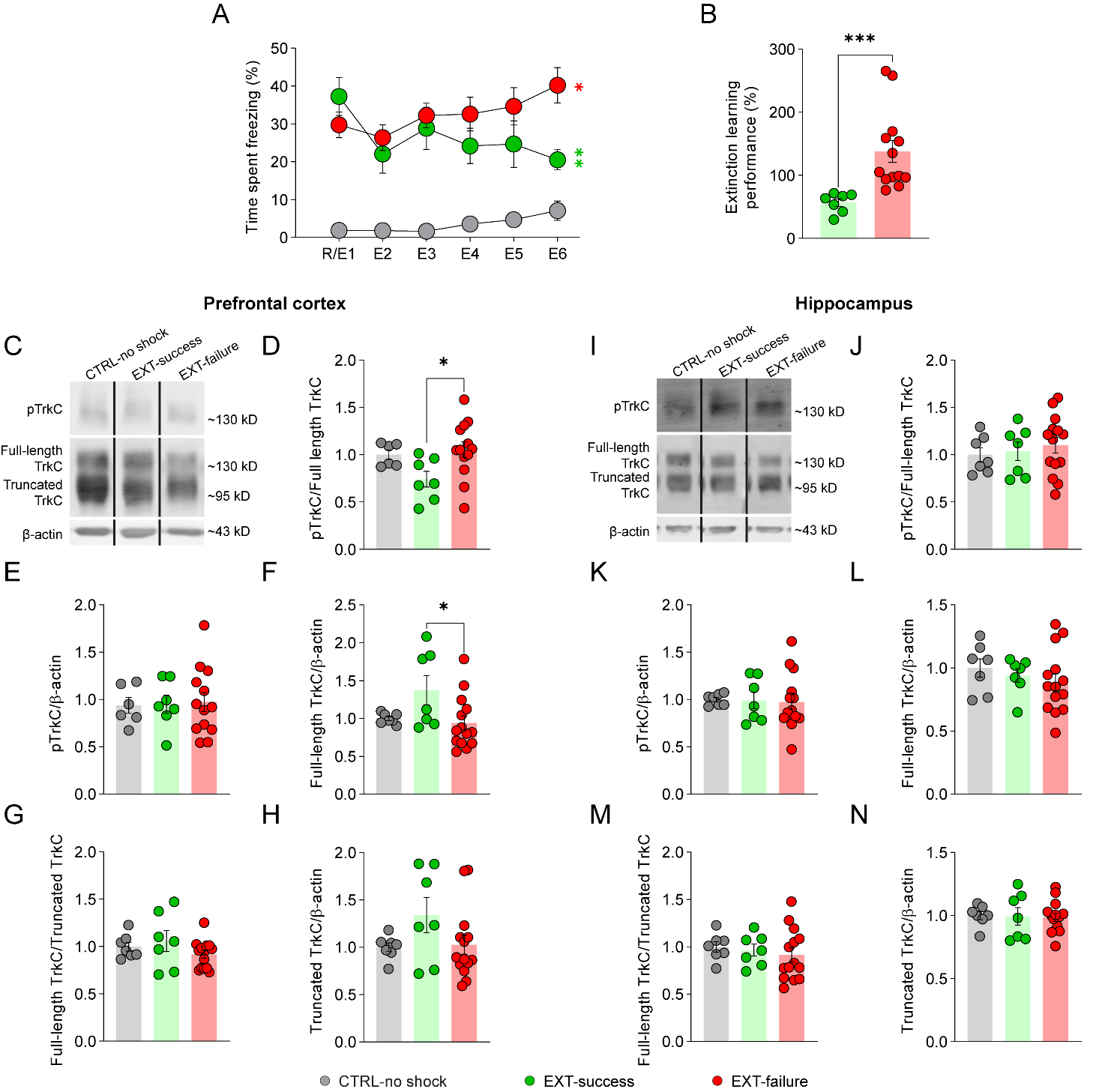
**Fig. S4. Fear extinction performance of mice used in western blot experiments. (A)** Quantification of the percentage of time spent freezing during extinction acquisition of fear conditioned mice. EXT-success (n = 7) mice significantly decreased their freezing levels from R/E1 to E6, while EXT-failure (n = 14) further increased their freezing levels (repeated measures two-way ANOVA, extinction trial x group interaction F_(10, 125)_ = 2.656, p = 0.0057; Sidak multiple comparisons test, EXT-success, R/E1 vs. E6, t = 3.572, p = 0.0075; EXT-failure, R/E1 vs. E6, t = 3.160, p = 0.0292). **(B)** Quantification of the ELP ratio of fear conditioned mice trained in the extinction paradigm. Mice were categorized as EXT-success or EXT-failure according to their ELP. The dotted line marks the threshold of 30% reduction in freezing, used to categorize mice as EXT-success (Mann-Whitney U test, U = 0, p < 0.001). **(C, I)** Representative images of western blots for pTrkC, full-length TrkC and truncated TrkC performed in brain extracts from the **(C)** PFC and **(I)** hippocampus. Each panel shows non-contiguous lanes from the same membrane. Quantification of full length and truncated TrkC levels and of full length TrkC activation in **(C-H**) the PFC and in **(J-N)** the hippocampus. **(D)** EXT-success mice showed lower pTrkC/full-length TrkC ratio as compared with EXT-failure mice, but not with CTRL-no shock mice (Kruskal-Wallis H = 6.158, p < 0.0460; Dunn's multiple comparison test, EXT-success vs. EXT-failure Z = 2.433, p = 0.0449; CTRL-no shock vs. EXT-success Z = 1.740, p = 0.2455; CTRL-no shock vs. EXT-failure Z = 0.3498, p > 0.9999). **(E)** No differences were observed in total pTrkC levels among groups (one-way ANOVA, F_(2, 23)_ = 0.03432, p = 0.9663). **(F)** EXT-success mice showed higher full-length TrkC levels as compared with EXT-failure mice, but not with CTRL-no shock mice (one-way ANOVA F_(2, 25)_ = 3.591, p = 0.0426; Sidak multiple comparisons test, EXT-success vs. EXT-failure t = 2.623, p = 0.0433; CTRL-no shock vs. EXT-success t = 1.964, p = 0.1177; CTRL-no shock vs. EXT-failure t = 0.3543, p = 0.7261). In what respects the TrkC truncated isoform, no differences were observed among groups in **(G)** full-length TrkC/truncated TrkC ratio (one-way ANOVA, F _(2, 25)_ = 1.438, p = 0.2563) or **(H)** truncated TrkC levels (one-way ANOVA, F _(2, 25)_ = 2.011, p = 0.1550). Overall, in the hippocampus no differences were observed among groups in **(J)** the pTrkC/full-length TrkC ratio (one-way ANOVA, F_(2, 25)_ = 0.3469, p = 0.7102), **(K)** total pTrkC levels (one-way ANOVA, F_(2, 25)_ = 0.02785, p = 0.9726), **(L)** total full-length TrkC levels (one-way ANOVA, F_(2, 25)_ = 0.6150, p = 0.5486), **(M)** full-length TrkC/truncated TrkC ratio (one-way ANOVA, F _(2, 25)_ = 0.3700, p = 0.6944) and **(N)** truncated TrkC levels (one-way ANOVA, F _(2, 25)_ = 2.938, p = 0.0714). E1 to E6, extinction trials; ELP, extinction learning performance; PFC, prefrontal cortex; pTrkC, phosphorylated TrkC; R, fear retrieval; TrkC, tropomyosin receptor kinase C *p ≤ 0.05, **p ≤ 0.01, ***p ≤ 0.001.


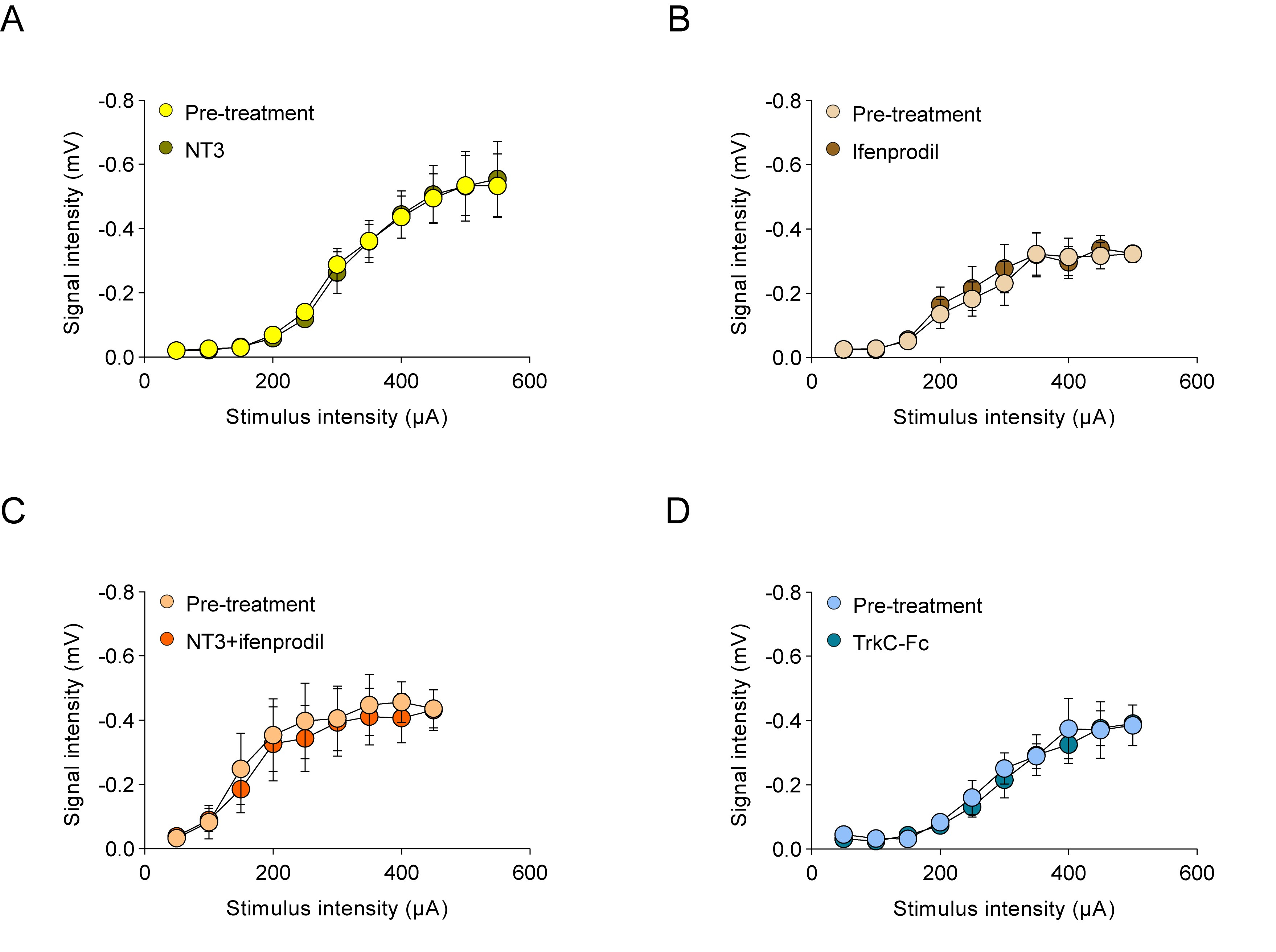


**Fig. S5. I/O curves of electrophysiology experiments performed in LA-containing slices treated pharmacologically.** I/O curves were obtained before and 30 min after incubation with **(A)** NT3, **(B)** ifenprodil, **(C)** NT3+ifenprodil and **(D)** TrkC-Fc. NT3 (n = 6), ifenprodil (n = 4) and NT3+ifenprodil (n = 5) were applied to EXT-failure slices; TrkC-Fc (n = 4) was applied to EXT-success slices. None of the treatments affected the I/O curves (repeated measures two-way ANOVA, stimulus x treatment interaction; NT3, F_(10, 100)_ = 0.04059, p > 0.9999; ifenprodil, F_(9, 54)_ = 0.2297, p = 0.9887; NT3+ifenprodil, F_(8, 64)_ = 0.1726, p = 0.9938; TrkC-Fc, F_(9, 54)_ = 0.1879, p = 0.9946). I/O, input/output; PS, population spike.
